# Advancements in Immunotherapy for Lung Cancer: A Meta‐Analysis of Clinical Trial Outcomes

**DOI:** 10.1111/crj.70223

**Published:** 2026-09-09

**Authors:** Yingjie Jiao, Yan Zhao

**Affiliations:** ^1^ Boshan Shantou Health Center Zibo China; ^2^ Department of Cadiothoracic Surgery Zibo First Hospital Zibo China

**Keywords:** chemo‐immunotherapy, immune checkpoint inhibitors, non–small cell lung cancer, overall survival, progression‐free survival

## Abstract

Immune checkpoint inhibitors (ICIs) have changed therapeutic selections for advanced non–small cell lung cancer (NSCLC). However, comparative evidence versus chemotherapy through ICI monotherapy and chemo‐immunotherapy combination strategies remains incomplete. For this purpose, we conducted a meta‐analysis of randomized controlled trials (RCTs) to measure overall survival (OS) and progression‐free survival (PFS) outcome improvements in ICI monotherapy or ICI‐chemotherapy combined treatment. These interventions take place in adults (≥ 18 years) with advanced/metastatic NSCLC. The Preferred Reporting Items for Systematic Reviews and Meta‐Analyses directions were obeyed. PubMed, Scopus, and Web of Science were searched (2021–2024) for RCTs. A total of 7849 articles were retrieved. RCTs (*n* = 17) qualify inclusion. Hazard ratios (HRs) along with 95% confidence intervals (CIs) for OS and PFS were pooled using random‐effects models. The heterogeneity was assessed by *I* ^2^. Risk of bias was valued with RoB 2. Publication bias and leave‐one‐out sensitivity analyses were also estimated. ICI monotherapy RCTs (*n* = 5) significantly enhanced OS (pooled HR = 0.73, 95% CI: 0.64–0.85; *p* < 0.0001) and PFS (pooled HR = 0.69, 95% CI: 0.49–0.96) with increased heterogeneity (*I* ^2^ = 90%). Combined ICI‐chemotherapy RCTs (*n* = 12) produced great benefits, with a pooled OS (HR = 0.68, 95% CI: 0.61–0.75; *p* < 0.00001; *I* ^2^ = 48%) and pooled PFS (HR = 0.55, 95% CI: 0.50–0.61; *p* < 0.00001; *I* ^2^ = 56%). Subgroup analyses proposed durable effects in smaller trials and with pembrolizumab‐based treatments. Risk‐of‐bias valuations underlined concern, primarily for deviations from therapeutic interventions. Sensitivity analyses set robustness, whereas publication bias was limited. ICI‐based remedies, especially chemo‐immunotherapy, significantly improve OS and PFS. Heterogeneity and experimental‐level bias warrant cautious interpretation. Hence, large RCTs are important to choose the best treatment possibility.

## Graphical abstract

The graphical abstract presents a meta‐analysis of randomized controlled trials (RCTs) associating immune checkpoint inhibitors (ICIs) with or without chemotherapy versus chemotherapy alone for adults with advanced or metastatic non–small cell lung cancer (NSCLC)

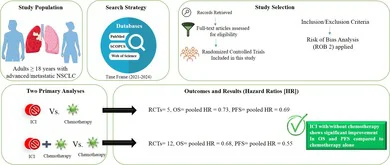

## 1 Introduction

Lung cancer is the primary cause of cancer‐related death worldwide, with non–small cell lung cancer (NSCLC) making for nearly 85% of all cases [1]. For a long time, NSCLC has been detected with a grave prognosis and fewer treatment options than afore. The 5‐year survival rate for NSCLC remains < 18%, with over half of patients dying within 1 year of diagnosis. This poor prognosis is predominantly apparent in advanced/metastatic disease, where the goal of therapy changes from therapeutic to palliative care [1]. Traditionally, platinum‐based chemotherapy has been the regular first‐line treatment for advanced NSCLC, yet response rates are limited, and median overall survival (OS) is roughly 8–12 months [2]. Besides, patients who progress after initial chemotherapy feel even worse consequences, with docetaxel monotherapy providing modest advantages in the second‐line setting [3, 4]. These confinements accentuated the burning need for novel therapeutic methods that could enhance survival outcomes and quality of life. With the introduction and inclusion of immune checkpoint inhibitors (ICIs), significant developments have been taken place in therapeutic patterns for NSCLC [5, 6, 7]. Currently, immunotherapy, especially ICIs, has increased its effectiveness by targeting the programmed death‐1/programmed death‐ligand 1 (PD‐1/PD‐L1) signaling pathway and stalling the inhibitory mechanisms that allow tumor cells to evade immune recognition. Under normal conditions, the PD‐1/PD‐L1 interaction inhibits T‐cell activation. This allows tumor cells to live within the host and go unnoticed by the immune system. ICIs interrupt this interaction and reinstate T‐cell function. Hence, they enable the immune system to identify and eradicate cancer cells. This mechanism has extraordinarily upgraded OS and progression‐free survival (PFS) compared to traditional chemotherapy [5, 6, 7]. The ICIs, including pembrolizumab, nivolumab, atezolizumab, and others, open up an immune‐centric cure strategy for lung cancers apart from chemotherapy [8, 9, 10]. These agents have shown superior efficacy through different NSCLC subtypes, regardless of PD‐L1 expression levels, though responses are largely more prominent in patients with higher PD‐L1 expression. The clinical efficacy of ICIs in NSCLC was first recognized in phase III studies comparing nivolumab with docetaxel in patients who had advanced after platinum‐based chemotherapy. These studies, like CheckMate 057 and CheckMate 017, confirmed that nivolumab improved OS and had a higher objective response rate (ORR). This has positioned it as a prominent option in the treatment regimen for second‐line therapy. Succeeding trials proved pembrolizumab as a first‐line preference for patients with PD‐L1 expression ≥ 1%, which has further expanded the continuum of immunotherapy [8, 9, 10]. Immunotherapeutic tactics apart from PD‐1/PD‐L1 inhibitors are presently under development. These include CTLA‐4 inhibitors, LAG‐3 inhibitors, and novel combination approaches. Till now, combination remedies (ICIs + chemotherapy) have shown potential in refining therapeutic effects [11, 12]. In spite of these developments, several serious questions remain unrequited. These include the best sequencing of ICI monotherapy versus combination therapy, the comparative efficiency in diverse PD‐L1 expression subgroups, and the long‐standing safety profiles that entail further investigation. In addition, although some meta‐analyses have inspected specific ICI agents or cure lines, widespread valuations matching ICI monotherapy, ICI‐chemotherapy combinations, and standard chemotherapy through all treatment situations are restricted [13]. Keeping the about points in consideration, the current meta‐analysis was designed with aims to evaluate the effects of ICIs with or without chemotherapy versus standard chemotherapy on survival outcomes in adults with advanced/metastatic NSCLC. Specifically, we addressed whether (1) ICI monotherapy improves OS and PFS compared with chemotherapy alone, and (2) ICI combined with chemotherapy improves OS and PFS compared with chemotherapy alone. These questions are addressed through a meta‐analysis of randomized controlled trials, the methodology for which is described in the following section. Our findings will provide crucial evidence to guide clinical decision‐making and optimize treatment strategies for patients with advanced NSCLC.

## 2 Methodology

### 2.1 Search Strategy

This meta‐analysis was performed according to the Preferred Reporting Items for Systematic Reviews and Meta‐Analyses (PRISMA) guidelines and also kept the methodological commendations from the Cochrane Handbook for Systematic Reviews of Interventions [14, 15]. An extensive and reproducible search strategy was used to identify studies that analyzed the impact of ICIs with or without standard chemotherapy versus chemotherapy in NSCLC. The search strategy consisted of controlled vocabulary terms (MeSH), free‐text keywords, and Boolean operators (“AND” and “OR”) and field tags specific to the database were used. Three major databases, such as PubMed, Scopus, and Web of Science (WOS), were searched. The comprehensive search strategies are given in the Supporting Information. The search was restricted to studies from 2021 to 2024. Following initial identification, screening was conducted to evaluate the relevance of the identified studies. Screening looked more particularly in studies where direct comparisons were made between the selected ICIs with or without chemotherapy versus chemotherapy for a thorough analysis to extract quantitative data for outcomes (OS and PFS) in NSCLC patients. All retrieved records were exported into EndNote reference management software, where duplicate entries were identified and removed prior to the screening process [16, 17].

### 2.2 Eligibility Criteria

We only included studies that were randomized clinical trials (RCTs) using ICI treatment (e.g., pembrolizumab or nivolumab or atezolizumab) or alongside chemotherapy (e.g., pembrolizumab plus carboplatin/pemetrexed), and these were compared against chemotherapy alone in adults ≥ 18 years old with advanced/metastatic NSCLC. The studies needed to deliver enough statistical information, which would allow for meta‐analysis of survival results via hazard ratio (HR) measurements. This included 95% confidence intervals (CIs), standard error (SE) values, variance determinations, *p*‐value results, and log (HR) measurements with their SE values. On the other hand, studies were excluded if participants were < 18 years old or if they had locally advanced/metastatic NSCLC and SCLC. Furthermore, studies were also excluded if interventions in studies lacked ICI monotherapy or ICI plus chemotherapy, comparators were not chemotherapy alone, study designs were nonrandomized trials (observational studies, reviews, abstracts without full text, meta‐analysis, and systematic review), and insufficient statistical data, such as HR, 95% CI, SE, OS, and PFS, were described.

### 2.3 Screening Criteria

Screening was performed in two stages. In Stage 1, titles and abstracts were screened using Rayyan AI [18] by two independent reviewers to identify and detect potentially eligible studies. In Stage 2, full‐text screening was executed independently by two reviewers, and each study was categorized rigorously as either include or exclude, with no maybe category.

### 2.4 Quality Analysis

Quality analysis was performed using the RoB 2 tool [19]. Two reviewers independently assessed risk of bias for the survival outcomes of OS and PFS across five domains, which were randomization process, deviations from intended interventions, missing outcome data, outcome measurement, and selection of the reported result. Information was extracted only from the full text, protocol, trial registration, statistical analysis plan, tables, figures, and supplementary materials when available. No assumptions were made about missing information. For each study, a combined study‐level judgment was assigned across OS and PFS using a conservative approach, where the higher risk judgment was selected when outcome‐specific information differed. Overall risk of bias was classified as low risk, some concerns, or high risk according to RoB 2 criteria. Visualizations of risk‐of‐bias assessments were produced using robvis (Risk‐Of‐Bias VISualization) [20].

### 2.5 Data Extraction

Data extraction was performed in two steps. In the first step, study characteristics were extracted for all the included studies. Then, in the second step, data extraction for meta‐analysis was performed by two independent reviewers. Outcome‐level survival data were extracted independently from each eligible study using a predefined extraction form. For each study, data were extracted only for the overall/intention‐to‐treat population and for eligible comparisons of ICI monotherapy or ICI plus chemotherapy versus chemotherapy alone. Extracted variables included study ID, sample size, NSCLC stage, ICI drug, HR, lower and upper 95% CIs, log (HR), and SE. HRs and CIs were extracted exactly as stated in the full text, whereas log (HR) was calculated as the natural logarithm of the reported HR. Only eligible survival outcomes, including OS and PFS, were extracted. Whereas subgroup‐specific results, response outcomes, adverse events, quality‐of‐life outcomes, and biomarker‐only data were not extracted.

### 2.6 Statistical Analysis

Statistical analysis was performed using the random‐effects model (REML) in Review Manager (RevMan) [21]. HRs with 95% CIs were pooled for OS and PFS outcomes. Meta‐analyses were conducted separately for two predefined comparisons: (1) ICI monotherapy versus chemotherapy alone and (2) ICI plus chemotherapy versus chemotherapy alone. For quantitative synthesis, HRs were transformed into log (HR), and SEs were calculated from the reported 95% CIs using formula:

$$\mathrm{SE}=\left[\ln\;\left(\text{upper}\;\mathrm{CI}\right)-\ln\;\left(\text{lower}\;\mathrm{CI}\right)\right]/3.92$$

Statistical heterogeneity was assessed using the *I* ^2^ statistic. Prespecified subgroup analyses were performed according to population size, ICI drug subgroup, and NSCLC stage using RevMan [20]. Publication bias was assessed in R using funnel plot analysis and Egger's regression test. Sensitivity analysis was performed using the leave‐one‐out method to evaluate the influence of individual studies on the pooled effect estimates [22, 23, 24].

## 3 Results

### 3.1 Study Selection

**FIGURE 1:**
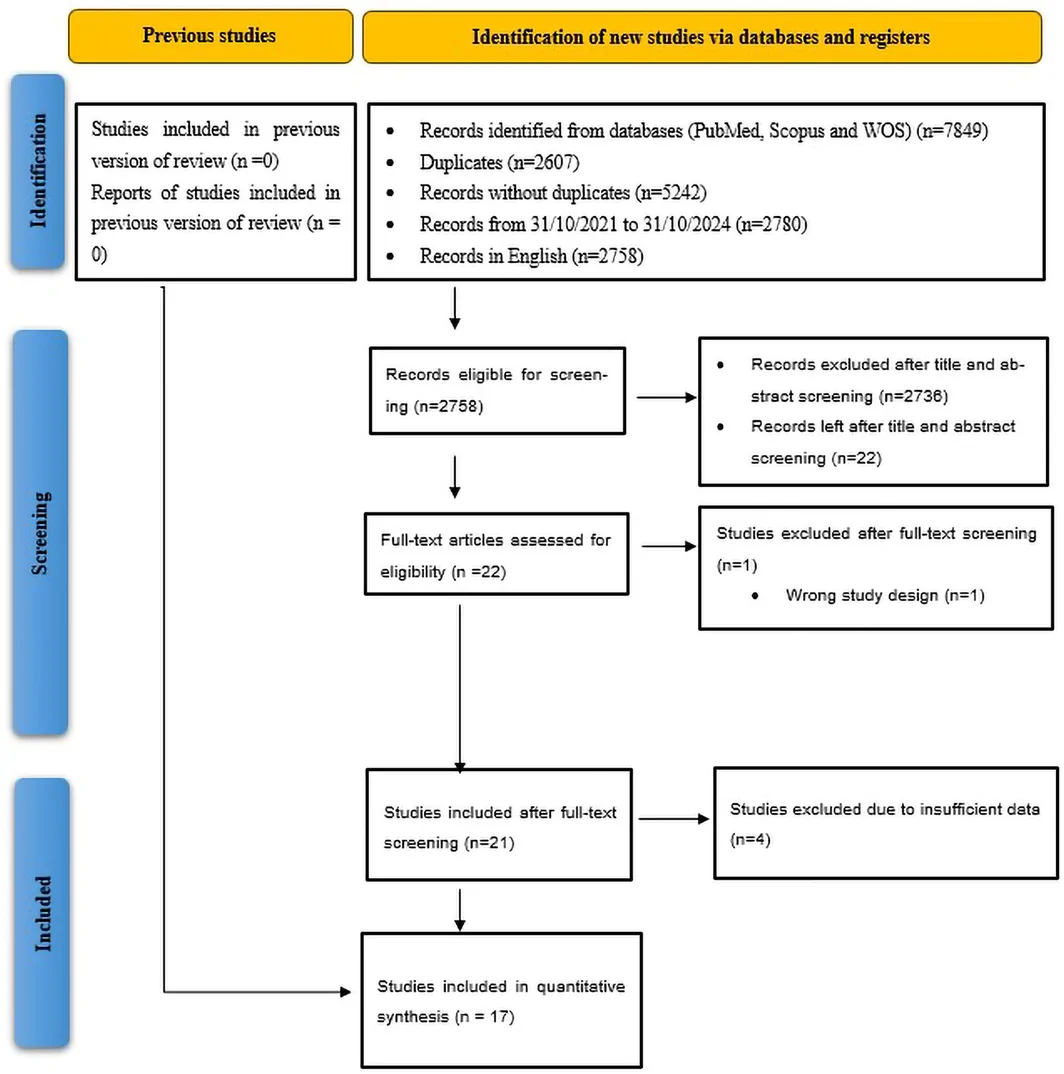
PRISMA flow diagram for the ICI with or without chemotherapy versus chemotherapy meta‐analysis. This figure illustrates the flow of information through different phases such as records, screening, and eligible studies.

The study selection process was performed following with PRISMA 2020 guidelines [14]. A total of 7849 records were retrieved through database searching, including 1120 from PubMed, 4312 from Scopus, and 2417 from WOS. After the removal of 2607 duplicate records using EndNote as reference management software [16, 17], 5242 unique studies stayed. Among these, 22 studies published in languages other than English were excluded. This has resulted in 2758 records eligible for title and abstract screening. During title and abstract screening using Rayyan AI [18], 2736 records were excluded based on predefined eligibility criteria, leaving 22 studies for further consideration. One study, Fan et al. [25], excluded during full‐text screening due to wrong study design, and four studies [26, 27, 28, 29] were excluded due to insufficient statistical data. Ultimately, 17 studies met all inclusion criteria and were included in the final quantitative synthesis. The complete study selection process is presented in the PRISMA flow diagram (Figure 1).

### 3.2 Study Characteristics

The characteristics of the included studies are summarized in Table 1. These 17 RCTs evaluated first‐line immunotherapy‐based regimens with or without chemotherapy versus chemotherapy alone in advanced NSCLC, with a combined methodical population of 8023 patients. Studies varied in design (Multicenter Phase III, open‐label, and double‐blind), sample size, histology (squamous vs. nonsquamous), PD‐L1 selection (tumor proportion score (TPS) ≥ 1% or ≥ 50%), ICIs used (pembrolizumab or nivolumab or nivolumab‐ipilimumab or atezolizumab), and chemotherapy backbones. Comprehensive study‐level information is given in Table 1.

**TABLE 1:** Study characteristics of included studies.

References	Country	Study design/phase	Total sample size	Population	Disease stage	Histology	Line of therapy	Intervention	Comparator	Survival outcomes reported
Awad et al. [58]		Randomized 1:1	123	Adult patients with Stage IIIB/IV nonsquamous NSCLC without sensitizing EGFR or ALK alterations, no previous systemic treatment for NSCLC or untreated brain metastases, and with tumor samples for PD‐L1 assessment	Stage IIIB/IV	Nonsquamous NSCLC	No previous systemic treatment for NSCLC	Pembrolizumab 200 mg plus pemetrexed 500 mg/m^2^ and carboplatin at area under the concentration‐time curve of 5 mg/mL/min every 3 weeks for four cycles, followed by pembrolizumab 200 mg every 3 weeks for up to an additional 31 cycles or a total of 2 years with optional pemetrexed maintenance therapy	Chemotherapy alone	PFS, OS
Borghaei et al. [59]	North America; Europe; Asia; Rest of the world	Open‐label, randomized, phase III study	755	Eligible patients were aged ≥ 18 years with metastatic (Stage IV or recurrent), histologically confirmed nonsquamous or squamous NSCLC and an Eastern Cooperative Oncology Group performance status of 0 or 1 who had not received any prior systemic anticancer treatment as primary therapy for advanced disease	Metastatic (Stage IV or recurrent)	Histologically confirmed nonsquamous or squamous NSCLC	First‐line	Nivolumab 360 mg every 3 weeks plus histology‐based platinum‐doublet chemotherapy	Histology‐based platinum‐doublet chemotherapy	OS; PFS
Carbone et al. [60]	International	Randomized, open‐label, international phase 3 trial	719	Patients were aged ≥ 18 years with squamous or nonsquamous Stage IV/recurrent NSCLC, Eastern Cooperative Oncology Group performance status 0 or 1, and no known sensitizing EGFR/ALK alterations	Stage IV/recurrent NSCLC	Squamous or nonsquamous	NR	Nivolumab (360 mg every 3 weeks) plus ipilimumab (1 mg/kg every 6 weeks) combined with platinum‐doublet chemotherapy (every 3 weeks for two cycles)	Chemotherapy alone (every 3 weeks for four cycles); optional pemetrexed maintenance therapy (500 mg/m^2^ every 3 weeks), permitted for the nonsquamous population of the control arm only	OS; PFS
Cheng et al. [61]	Mainland China	Global, randomized, placebo‐controlled, double‐blind, phase 3 KEYNOTE‐407 study; China extension study	125	Patients were at least 18 years of age with histologically or cytologically confirmed diagnosis of Stage IV squamous NSCLC, measurable disease per RECIST version 1.1, ECOG performance status of 0 or 1, adequate organ function, and not received systemic treatment previously	Stage IV	Squamous NSCLC	Not received systemic treatment previously	Pembrolizumab 200 mg every 3 weeks plus carboplatin plus paclitaxel	Saline placebo plus carboplatin plus paclitaxel	OS, PFS
de Castro Jr. et al. [62]	International	Multicenter, international, randomized, open‐label, controlled trial; Phase III	1274	PD‐L1 positive (TPS ≥ 1%), previously untreated advanced or metastatic non–small cell lung cancer (NSCLC)	Advanced or metastatic	Squamous vs. nonsquamous	Previously untreated	Pembrolizumab (MK‐3475) 200 mg intravenous (IV) every 3 weeks (Q3W)	SOC: investigator's choice of carboplatin AUC 5 or 6 + paclitaxel 200 mg/m^2^ Q3W for a maximum of 6 cycles, followed by optional pemetrexed 500 mg/m^2^ for subjects with nonsquamous histologies; or carboplatin AUC 5 or 6 + pemetrexed 500 mg/m^2^ Q3W for a maximum of 6 cycles, followed by optional pemetrexed 500 mg/m^2^ for subjects with nonsquamous histologies	OS; PFS
Garassino et al. [63]	NR	Randomized, open‐label, phase III study	1175	Adult patients with previously untreated locally advanced/metastatic NSCLC and PD‐L1 TPS ≥ 1% without sensitizing EGFR mutation or ALK translocation	Locally advanced/metastatic	Advanced squamous and nonsquamous NSCLC	Previously untreated	Pembrolizumab 200 mg every 3 weeks for 35 cycles	Investigator's choice of carboplatin AUC 5–6 mg/mL/min plus paclitaxel 200 mg/m^2^ or pemetrexed 500 mg/m^2^ (nonsquamous histology only) every 3 weeks for 4–6 cycles, followed by optional maintenance therapy of pemetrexed 500 mg/m^2^ for patients with nonsquamous histology	OS, PFS
Garassino et al. [64]	Worldwide	Worldwide, randomized, active‐controlled, parallel‐group, multisite, double‐blind trial	616	Subjects with advanced or metastatic nonsquamous non–small cell lung cancer (NSCLC) who have not previously received systemic therapy for advanced disease and in whom EGFR or ALK‐directed therapy is not indicated	Advanced or metastatic	Nonsquamous non–small cell lung cancer (NSCLC)	First line (1 L); have not previously received systemic therapy for advanced disease	Pembrolizumab 200 mg + pemetrexed 500 mg/m^2^ (with vitamin supplementation) + cisplatin 75 mg/m^2^ OR carboplatin AUC 5, all on Day 1 every 3 weeks (Q3W) for 4 cycles followed by pembrolizumab 200 mg + pemetrexed 500 mg/m^2^ Q3W until progression	Saline placebo + pemetrexed 500 mg/m^2^ (with vitamin supplementation) + cisplatin 75 mg/m^2^ OR carboplatin AUC 5, all on Day 1 Q3W for 4 cycles followed by saline placebo + pemetrexed 500 mg/m^2^ Q3W until progression	OS, PFS
Horinouchi et al. [65]	Japan	Phase 3, global, randomized, double‐blind, placebo‐controlled KEYNOTE‐189 trial; Japan extension study design was identical, with the exception that it only enrolled patients in Japan	40	Eligible patients had untreated Stage IV nonsquamous NSCLC without sensitizing EGFR or ALK alterations	Stage IV	Nonsquamous NSCLC	Untreated	Intravenous pembrolizumab 200 mg every 3 weeks plus pemetrexed 500 mg/m^2^ plus investigator's choice of cisplatin 75 mg/m^2^ or carboplatin area under the concentration–time curve 5 mg/mL/min every 3 weeks for four cycles, followed by maintenance therapy with pemetrexed 500 mg/m^2^ every 3 weeks	Saline placebo every 3 weeks plus pemetrexed 500 mg/m^2^ plus investigator's choice of cisplatin 75 mg/m^2^ or carboplatin area under the concentration–time curve 5 mg/mL/min every 3 weeks for four cycles, followed by maintenance therapy with pemetrexed 500 mg/m^2^ every 3 weeks	OS, PFS
Jassem et al. [66]	Europe; Asia Pacific; South America; North America	Open‐label, randomized, phase 3 trial	572	Patients with chemotherapy‐naive Stage IV nonsquamous or squamous NSCLC and measurable disease per the Response Evaluation Criteria in Solid Tumors version 1.1	Stage IV	Nonsquamous or squamous NSCLC	Chemotherapy‐naive	Atezolizumab monotherapy 1200 mg intravenously on day 1 of each 21‐day cycle	Platinum‐based chemotherapy; patients with nonsquamous disease received cisplatin 75 mg/m^2^ or carboplatin area under the curve 6 plus pemetrexed 500 mg/m^2^ for four or six cycles and then pemetrexed maintenance therapy; those with squamous NSCLC received cisplatin 75 mg/m^2^ plus gemcitabine 1250 mg/m^2^ or carboplatin area under the curve 5 plus gemcitabine 1000 mg/m^2^ for four or six cycles and then best supportive care	OS; PFS
Lu et al. [67]	Mainland China and Taiwan	Global randomized, open‐label, Phase III study	163	Patients with histologically or cytologically confirmed Stage IV nonsquamous NSCLC with measurable disease per RECIST version 1.1 who had an ECOG PS of 0 or 1 and had not received prior treatment for metastatic disease	Stage IV	Nonsquamous NSCLC	Had not received prior treatment for metastatic disease	Atezolizumab plus cisplatin plus pemetrexed; atezolizumab 1200 mg, cisplatin 75 mg/m^2^, pemetrexed 500 mg/m^2^; maintenance atezolizumab plus pemetrexed	Cisplatin plus pemetrexed; maintenance pemetrexed alone	Overall survival (OS); Progression‐free survival (PFS)
Nishio et al. [68]	North America, Western Europe, Eastern Europe, Latin America, and Asia	Randomized, phase 3, multicenter, open‐label study	578	Adults older than or equal to 18 years of age with histologically or cytologically confirmed Stage IV nonsquamous NSCLC	Stage IV	Nonsquamous NSCLC	Chemotherapy‐naive; no previous treatment for metastatic disease	Atezolizumab in combination with cisplatin or carboplatin plus pemetrexed; maintenance therapy with atezolizumab plus pemetrexed	Cisplatin or carboplatin plus pemetrexed; maintenance therapy with pemetrexed	OS, PFS
Nishio et al. [69]	Japan	Multicenter, randomized, open‐label, phase 3 trial	101	Japanese patients, both male and female and aged ≥ 18 years, with histologically or cytologically confirmed Stage IV NSCLC with measurable disease as defined by RECIST version 1.1	Stage IV	Nonsquamous NSCLC	First‐line treatment; chemotherapy‐naïve; no prior treatment for Stage IV nonsquamous NSCLC	Atezolizumab (1200 mg) plus pemetrexed (500 mg/m^2^) and, based on the investigator's choice, either cisplatin (75 mg/m^2^) or carboplatin (area under the curve of 6 mg/mL/min) on day 1 of a 3‐wk cycle, for 4 or 6 cycles; maintenance atezolizumab (1200 mg) plus pemetrexed (500 mg/m^2^)	Identical doses of pemetrexed and carboplatin or cisplatin; maintenance pemetrexed alone	Overall survival (OS); Progression‐free survival (PFS)
Novello et al. [37]	NR	Patients were randomly assigned 1:1	559	Previously untreated, histologically/cytologically confirmed Stage IV squamous NSCLC	Stage IV	Squamous NSCLC	Previously untreated	Pembrolizumab 200 mg once every 3 weeks plus carboplatin area under the curve 6 mg/mL/min plus paclitaxel 200 mg/m^2^ or nab‐paclitaxel 100 mg/m^2^ on days 1, 8, and 15 once every 3 weeks for four cycles, followed by pembrolizumab 200 mg	Placebo once every 3 weeks plus carboplatin area under the curve 6 mg/mL/min plus paclitaxel 200 mg/m^2^ or nab‐paclitaxel 100 mg/m^2^ on days 1, 8, and 15 once every 3 weeks for four cycles, followed by placebo	Overall survival (OS); Progression‐free survival (PFS)
Reck et al. [33]	East Asian or non–East Asian enrollment	Patients were randomly assigned (1:1)	305	Eligible patients were ≥ 18 years with previously untreated Stage IV NSCLC with PD‐L1 TPS of at least 50%, measurable disease by RECIST v1.1, Eastern Cooperative Oncology Group performance status of 0 or 1, and life expectancy of at least 3 months	Stage IV NSCLC	Squamous or nonsquamous	Previously untreated	200 mg of intravenous pembrolizumab once every 3 weeks for 35 cycles	Investigator's choice of platinum chemotherapy (carboplatin/cisplatin) plus pemetrexed or gemcitabine, or carboplatin plus paclitaxel for 4 to 6 cycles	Overall survival (OS); Progression‐free survival (PFS)
Rodríguez‐Abreu et al. [70]	NR	Randomized	616	Eligible patients were ≥ 18 years of age, with previously untreated histologically/cytologically confirmed Stage IV nonsquamous NSCLC, without EGFR/ALK aberrations	Stage IV	Nonsquamous NSCLC	Previously untreated	Pembrolizumab 200 mg intravenously every 3 weeks plus pemetrexed 500 mg/m^2^ and investigator's choice of either cisplatin 75 mg/m^2^ or carboplatin area under the curve 5 mg·min/mL for the first four cycles, followed by pemetrexed maintenance therapy	Saline placebo intravenously every 3 weeks plus pemetrexed 500 mg/m^2^ and investigator's choice of either cisplatin 75 mg/m^2^ or carboplatin area under the curve 5 mg·min/mL for the first four cycles, followed by pemetrexed maintenance therapy	Overall survival (OS); Progression‐free survival (PFS)
Satouchi et al. [71]	Japan	Prespecified subanalysis of the phase III, open‐label, randomized KEYNOTE‐024 study	40	Adult patients aged 18 years or older with previously untreated Stage IV NSCLC without activating EGFR mutations or ALK translocations, a PD‐L1 TPS of 50% or higher, measurable disease based on RECIST version 1.1, and an ECOG performance status of 0 or 1	Stage IV	Squamous vs. nonsquamous	Previously untreated	Pembrolizumab 200 mg i.v. every 3 weeks for up to 35 cycles	Investigator's choice of one of the following five platinum‐based chemotherapy regimens, selected before randomization, for four to six cycles: carboplatin or cisplatin plus pemetrexed, carboplatin or cisplatin plus gemcitabine, or carboplatin plus paclitaxel	Overall survival (OS); Progression‐free survival (PFS)
Wu et al. [72]	Mainland China	Global, prospective, randomized, open‐label, Phase 3 KEYNOTE‐042 study; KEYNOTE‐042 China extension study	262	Eligible patients enrolled from mainland China	NR	Squamous vs. nonsquamous histology	NR	Pembrolizumab 200 mg every 3 weeks (Q3W) for 35 cycles	Carboplatin AUC 5 or 6 plus investigator's choice of paclitaxel 200 mg/m^2^ or pemetrexed 500 mg/m^2^ Q3W for 4–6 cycles; optional pemetrexed maintenance encouraged for nonsquamous tumors	OS; PFS

### 3.3 Qualitative Analysis

**FIGURE 2:**
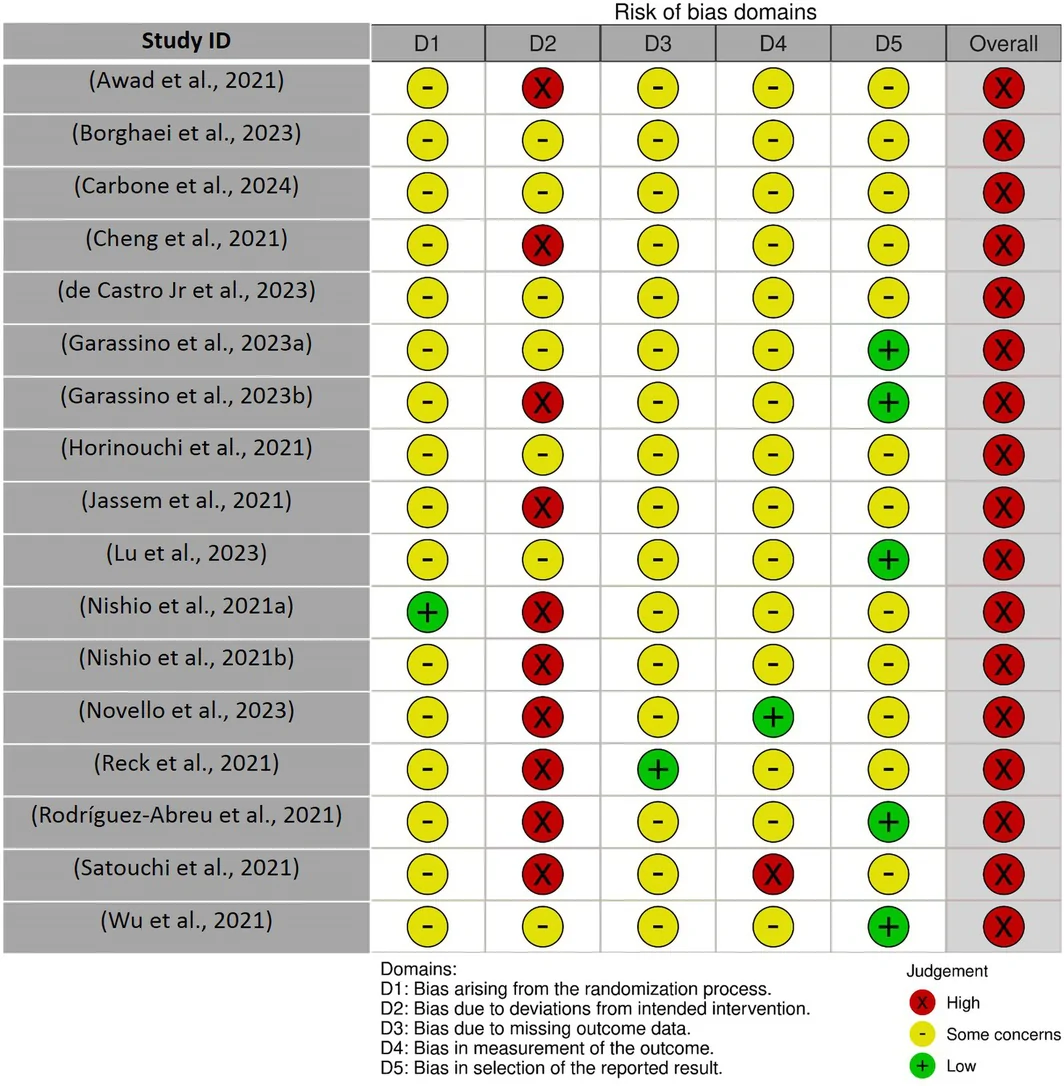
Traffic light plot, domain‐wise (D1–D5), showing levels of bias. Overall judgment is displayed alongside it too.

The risk‐of‐bias assessment throughout the 17 included studies, valued across five domains (D1–D5) and summarized as an overall judgment. This has indicated a generally high overall risk of bias for all trials, regardless of variable domain‐level evaluations (Figure 2). The traffic‐light plot obtained as a result of risk of bias analysis visualizes the patterns, with green (low risk) concentrated in D5, amber (some concerns) dominating D1, D3, and D4, and red (high risk) clustered mainly in D2. This has reflected systematic concerns about protocol adherence and outcome reporting throughout the body of evidence.

### 3.4 Quantitative Analysis

#### 3.4.1 Comparative Efficacy of ICI Monotherapy vs. Chemotherapy Alone

##### 3.4.1.1 OS Outcome

**FIGURE 3:**
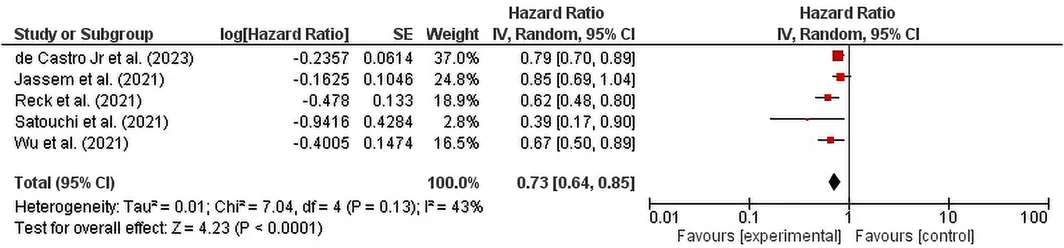
Forest plot comparing OS between ICI and chemotherapy. The rows list comprised studies/subgroups with reported log [HR], SE, study weight, and HRs with 95% CIs. The *x* axis is logarithmic from 0.01 to 100; the vertical line at HR = 1 indicates no difference between arms, and HR < 1 supports ICI. Pooled effect and heterogeneity statistics are also shown.

Quantitative synthesis using inverse‐variance random‐effects meta‐analysis of 5 RCTs showed a significant OS benefit with ICI monotherapy versus chemotherapy in patients with advanced NSCLC (pooled HR 0.73, 95% CI 0.64–0.85, *p* < 0.0001) as shown in Figure 3. Heterogeneity across studies was moderate (*I* ^2^ = 43%), and study weights ranged from 2.8% for Satouchi et al. [30] to 37% for de Castro et al. [31], with a significant overall effect size (*Z* = 4.23). As moderate between‐study heterogeneity and clinical differences across trials (e.g., sample size, immunotherapy agent, and disease stage) could impact the pooled estimate, we performed prespecified subgroup analyses to explore prospective effect modifiers and measure the robustness of the OS finding.

###### 3.4.1.1.1 Subgroup Analyses for OS

**FIGURE 4:**
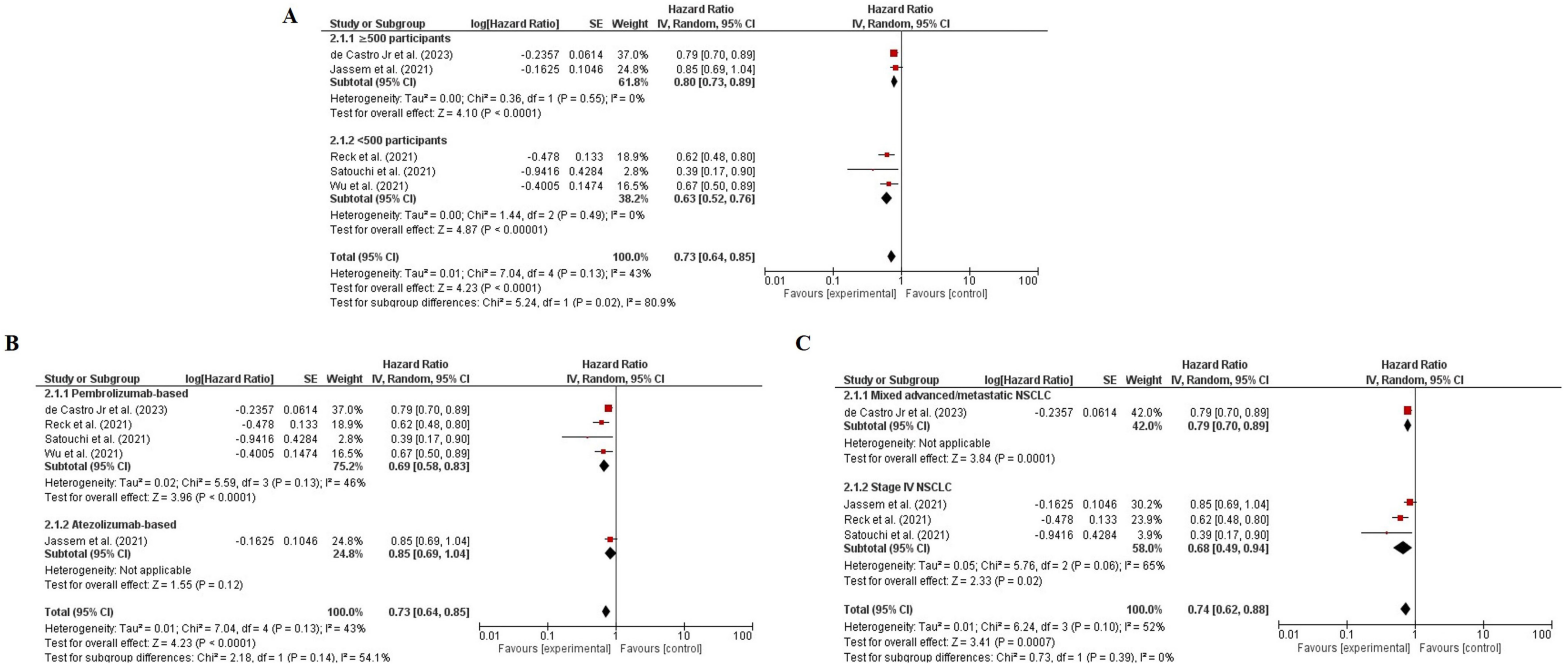
Forest plots of subgroup analyses comparing ICI versus chemotherapy for OS outcomes. HRs with 95% CIs (log [HR], SE, and study weight shown); *x*‐axis log scale 0.01–100 with 1 as the line of no effect. (A) By study size (≥ 500 vs. < 500), (B) by ICI agent (pembrolizumab‐based vs. atezolizumab‐based), and (C) by disease stage (mixed advanced/metastatic NSCLC vs. Stage IV NSCLC).

To study whether trial‐level characteristics modified the observed survival benefit, we conducted subgroup analyses of OS according to sample size, type of immunotherapeutic agent, and disease stage. Grouped by sample size, larger trials (≥ 500) in de Castro et al. [31] and Jassem et al. [32] studies reported a modest benefit (HR 0.80, 95% CI 0.73–0.89) versus smaller trials (< 500) conducted in Satouchi et al. [30], Reck et al. [33], and Wu et al. [34] studies, which showed a stronger effect (HR 0.63, 95% CI: 0.52–0.76), with a significant subgroup differences (*χ* ^2^ = 5.24, *p* = 0.02) suggesting potential small‐study effect (Figure 4A). By agent, pembrolizumab‐based trials in de Castro et al. [31], Reck et al. [33], Satouchi et al. [30], and Wu et al. [34] studies produced a greater benefit (HR 0.69, 95% CI: 0.58–0.83) than the single atezolizumab trial in Jassem et al. [32] study (HR 0.85, 95% CI: 0.69–1.04), though the differences were not significant (*χ* ^2^ = 2.18, *p* = 0.14) (Figure 4B). Lastly, both mixed advanced/metastatic trial in de Castro et al. [31] study and Stage IV–only trials in Jassem et al. [32], Reck et al. [33], and Satouchi et al. [30] studies showed survival benefits (HR 0.79, 95% CI: 0.70–0.89 and HR 0.68, 95% CI: 0.49–0.94, respectively) with no significant difference between stages (*χ* ^2^ = 0.73, *p* = 0.39) (Figure 4C). Taken together, these subgroup findings, though supportive of an OS benefit, suggest careful interpretation of larger effects from smaller trials. They further show no consistent modification of benefit by agent or disease stage, underlining the strength of the pooled result; however, they emphasize the need for confirmation in large and well‐powered studies.

###### 3.4.1.1.2 Publication Bias

**FIGURE 5:**
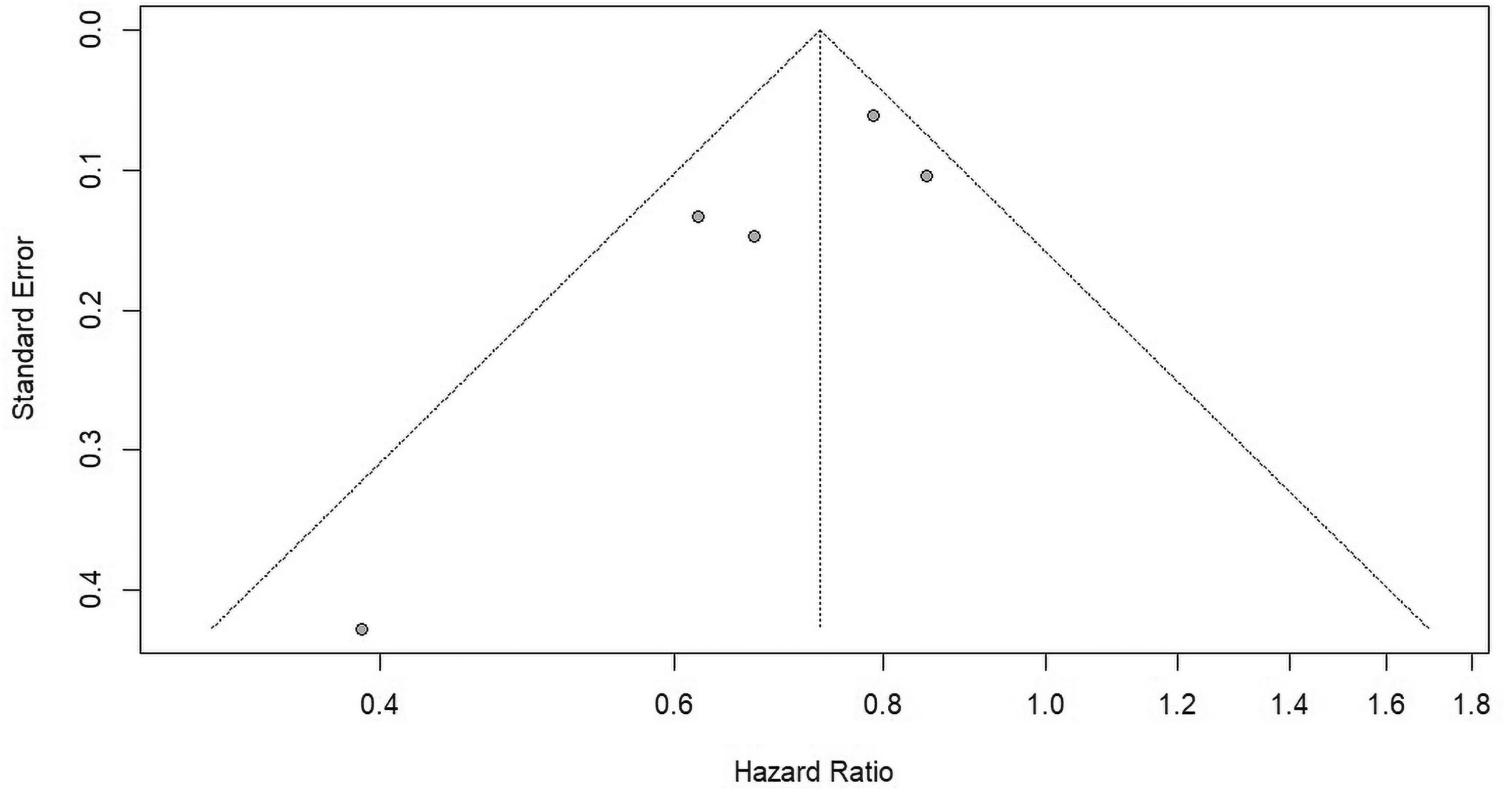
Funnel plot assessing publication bias for OS outcomes by comparing ICI versus chemotherapy, with HRs as the effect measure. Each dot represents an individual study. The *x* axis shows HRs, and the *y* axis shows SE.

Funnel plot analysis for publication bias displayed general symmetry through five studies, with log HRs ranging from approximately 0.4–0.88 and SEs from approximately 0.06–0.43 (Figure 5). Hence, no clear asymmetry proposed a low risk of bias, and yet limited studies disqualify formal tests like Egger's.

###### 3.4.1.1.3 Leave‐One‐Out Analysis

Leave‐one‐out sensitivity analysis validated high robustness of the pooled OS estimation (see the Supporting Information). In sequence exclusion of each study produced HRs ranging from 0.69 (95% CI: 0.5548–0.8593) omitting de Castro et al. [31] to 0.77 (95% CI: 0.6672–0.8847) omitting Reck et al. [33], whereas all remaining statistically significant (*p* < 0.001) and tightly gathered around the full‐model HR of 0.73 (95% CI: 0.6354–0.8466) (*p* < 0.0001). Heterogeneity was constantly moderate (*I* ^2^ = 31.9%–51.8%, *τ* ^2^ = 0.0057–0.0235), showing no single study improperly influenced the results, that is, de Castro et al. [31] (largest weight) exclusion generated the strongest effect and highest *τ* ^2^ (0.0235), whereas Reck et al. [33] removal produced the weakest HR but lowest heterogeneity, that is, *I* ^2^ = 31.9%.

##### 3.4.1.2 PFS Outcome

**FIGURE 6:**
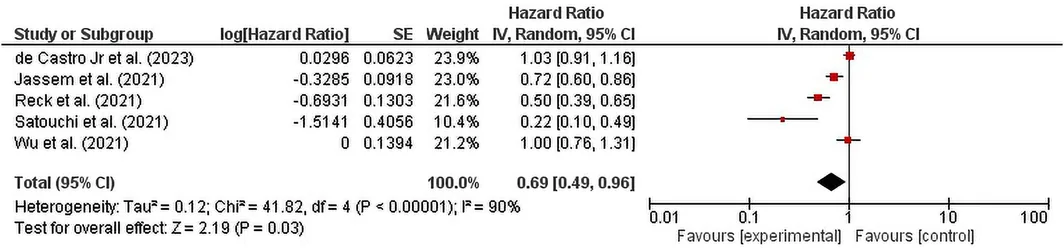
Forest plot associating ICI versus chemotherapy for PFS outcomes. The rows list encompassed studies/subgroups with described log [HR], SE, study weight, and HR with 95% CIs. The *x* axis, from 0.01 to 100, is logarithmic, whereas the vertical line at HR = 1 indicates no difference between arms, and the HR < 1 favors ICI. Pooled effect and heterogeneity statistics are also shown.

For PFS outcomes in NSCLC patients, five studies were included with HRs going from 0.22 to 1.03. The pooled HR was 0.69 (95% CI: 0.49–0.96), depicting a 31% reduction in the risk of disease progression or death among patients treated with ICIs monotherapy compared with chemotherapy. Among the included studies, de Castro et al. [31] reported a modest effect, that is, HR: 1.03 (95% CI: 0.91–1.16), whereas Jassem et al. [32] declared improved PFS with immunotherapy (HR: 0.72; 95% CI: 0.60–0.86). A favorable effect was described by Reck et al. [33], HR: 0.50 (95% CI: 0.39–0.65), and the most noticeable benefit was observed in the study by Satouchi et al. [30] (HR: 0.22; 95% CI: 0.10–0.49). In contrast, Wu et al. [34] showed no significant difference between treatment groups (HR: 1.00; 95% CI: 0.76–1.31). Significant heterogeneity was noted across studies (*I* ^2^ = 90%, *p* < 0.00001), suggesting significant inconsistency in study outcomes (Figure 6). However, the overall pooled analysis favors the dominance of ICIs over chemotherapy in improving PFS. Given the wide range of individual study effects, subgroup analyses were performed, as described in the following section, to evaluate study size, agent type, and disease stage impacts in the modification of PFS benefit of ICIs versus chemotherapy.

###### 3.4.1.2.1 Subgroup Analysis for PFS

**FIGURE 7:**
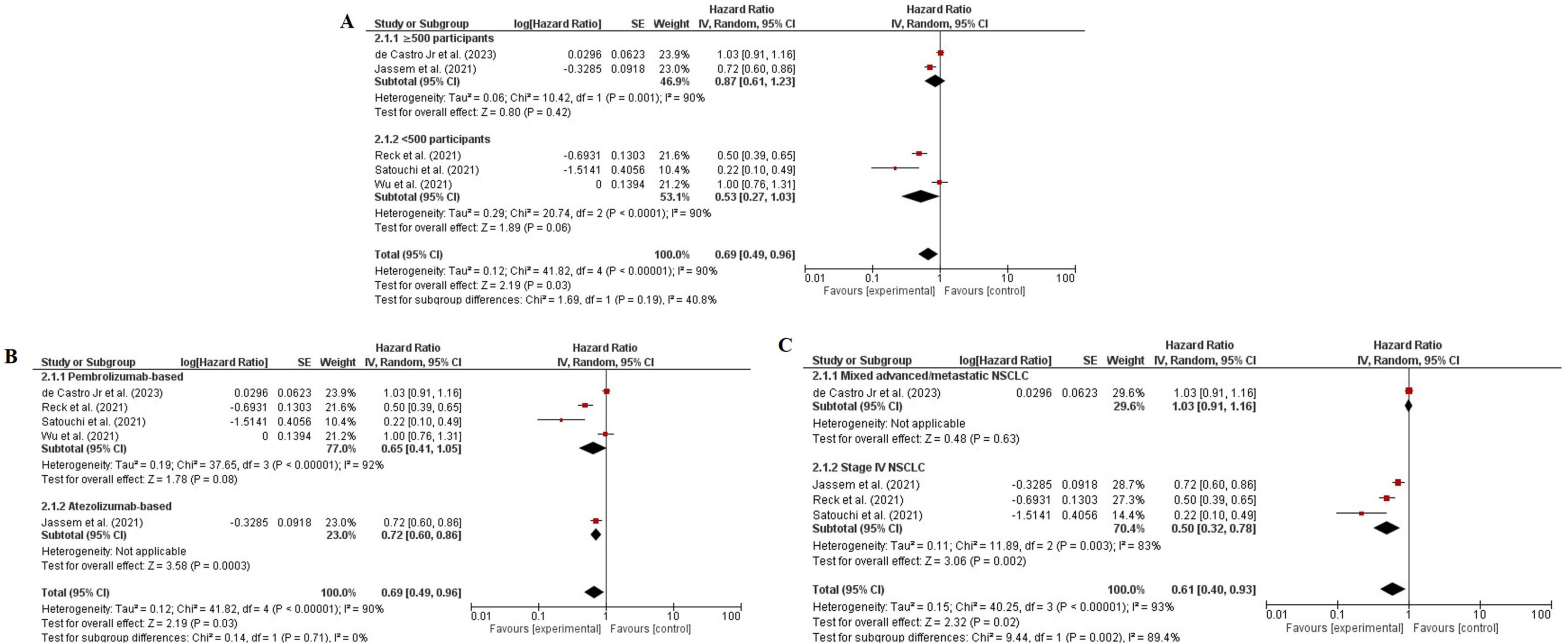
Forest plots of subgroup analysis comparing ICI versus chemotherapy for PFS outcomes. HRs with 95% CIs (log [HR], SE, and study weight shown); *x*‐axis log scale 0.01–100 with 1 as the line of no effect. (A) By study size (≥ 500 vs. < 500), (B) by ICI agent (pembrolizumab‐based vs. atezolizumab‐based), and (C) by disease stage (mixed advanced/metastatic NSCLC vs. Stage IV NSCLC).

Predefined subgroup analyses discovered sample size, immunotherapy agent, and disease stage to examine high between‐study heterogeneity, that is, overall *I* ^2^ = 93%. By sample size, large trials (≥ 500) [31, 32] showed no significant PFS benefit (HR 0.87 [95% CI: 0.61–1.23], *p* = 0.42; *I* ^2^ = 90%). On the other hand, smaller trials (< 500) [30, 33, 34] trended toward benefit (HR 0.53 [95% CI: 0.27–1.03], *p* = 0.06; *I* ^2^ = 90%). However, the subgroup test was not significant (*χ* ^2^ = 1.69, *p* = 0.19) (Figure 7A). By agent, pembrolizumab trials in de Castro et al. [31], Reck et al. [33], Satouchi et al. [30], and Wu et al. [34] studies showed a nonsignificant trend favoring pembrolizumab (HR 0.65 [95% CI: 0.41–1.05], *p* = 0.08; *I* ^2^ = 92%). On the other hand, the single atezolizumab trial [32] showed a significant PFS improvement (HR 0.72 [95% CI: 0.60–0.86], *p* = 0.0003). However, the agent differences were statistically nonsignificant (*χ* ^2^ = 0.14, *p* = 0.71) (Figure 7B). By stage, Stage IV–only RCTs performed in the studies of Jassem et al. [32], Reck et al. [33], and Satouchi et al. [30] confirmed a clear PFS advantage for ICIs (HR 0.50 [95% CI: 0.32–0.78]). In contrary, mixed advanced/metastatic RCT by de Castro et al. [31] did not, though overall heterogeneity remained high (*I* ^2^ = 93%) (Figure 7C). Hence, although ICIs improved PFS throughout the pooled dataset, high between‐study heterogeneity and nonsignificant subgroup interactions show that these subgroup findings should be interpreted carefully.

###### 3.4.1.2.2 Publication Bias

**FIGURE 8:**
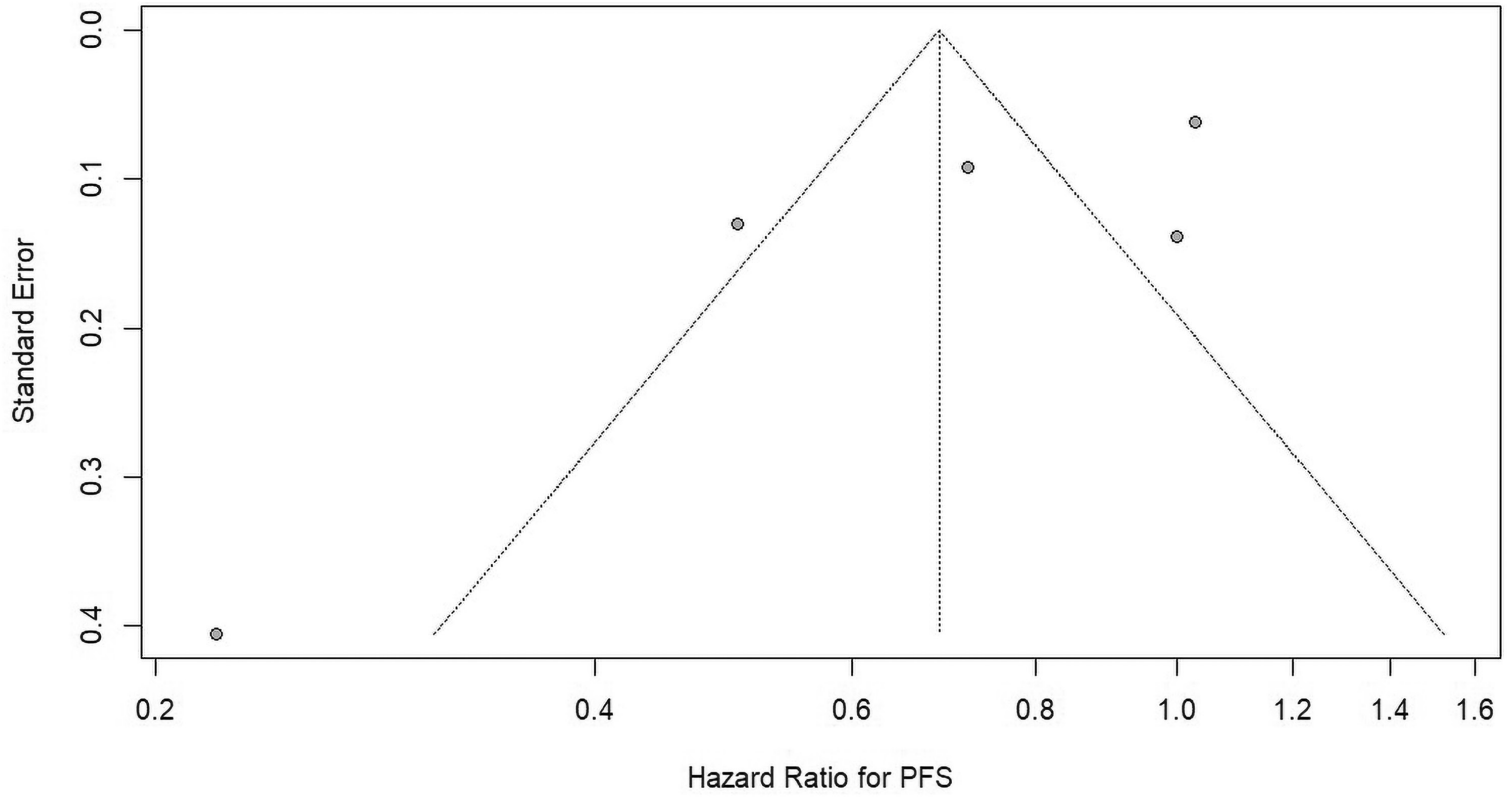
Funnel plot valuing publication bias analysis by comparing ICI versus chemotherapy for the PFS outcomes, with HRs as the effect measure. Each dot denotes a discrete study. The *x* axis shows HRs, and the *y* axis shows SE.

Funnel plot assessment showed approximate symmetry around the pooled effect and no obvious gap in the lower precision region (Figure 8). However, Egger's regression test for publication bias was not performed due to the limited number of studies (< 10). Hence, it prevents reliable detection of asymmetry. Although there was not any clear evidence of publication bias or small study effects, this analysis is also limited by the small number of RCTs.

###### 3.4.1.2.3 Leave‐One‐Out Analysis

Leave‐one‐out sensitivity analysis of the random‐effects meta‐analysis (pooled HR 0.6874 [95% CI: 0.4913–0.9618], *p* = 0.0287; *τ* ^2^ = 0.1192, *τ* = 0.3453, *I* ^2^ = 90.5%) revealed strong overall findings supporting ICI over chemotherapy. Statistical significance is reserved in most scenarios using the inverse variance method and DerSimonian‐Laird estimator for heterogeneity (Supporting Information). Excluding de Castro et al. [31] reinforced the effect (HR 0.6043 [95% CI: 0.4091–0.8928], *p* = 0.0114; *I* ^2^ = 86.0%), as did excluding Wu et al. [34] (HR 0.6109 [95% CI: 0.4029–0.9263], *p* = 0.0203; *I* ^2^ = 92.6%). Exclusion of Jassem et al. [32] bring forth a similar HR of 0.6536 (95% CI: 0.4088–1.0449, *p* = 0.0756; *I* ^2^ = 92.0%), approaching but not reaching significance. Removing Reck et al. [33] or Satouchi et al. [30] resulted in nonsignificant pooled effects (HR 0.7722 [95% CI: 0.5576–1.0694], *p* = 0.1197 and HR 0.7853 [95% CI: 0.5750–1.0725], *p* = 0.1286, respectively), along with high heterogeneity (*I* ^2^ = 87.1%–90.2%). These results specify that no single study overly drove the overall benefit, though high *I* ^2^ values throughout exclusions highlight considerable between‐study variability.

#### 3.4.2 Comparative Efficacy of ICI Monotherapy vs. Chemotherapy Alone

##### 3.4.2.1 OS Outcome

In the meta‐analysis of ICI plus chemotherapy versus chemotherapy alone for OS in NSCLC, HRs ranged from 0.29 to 0.86 across 12 studies, with resultant log (HR) values from −1.238 to −0.151 and SE from 0.0824 to 0.7147. SE values were deduced from the following formula:

$$\mathrm{SE}=\ln\;\left(\text{upper}\;\mathrm{CI}\right)-\ln\;\left(\text{lower}\;\mathrm{CI}\right)/3.92$$

**FIGURE 9:**
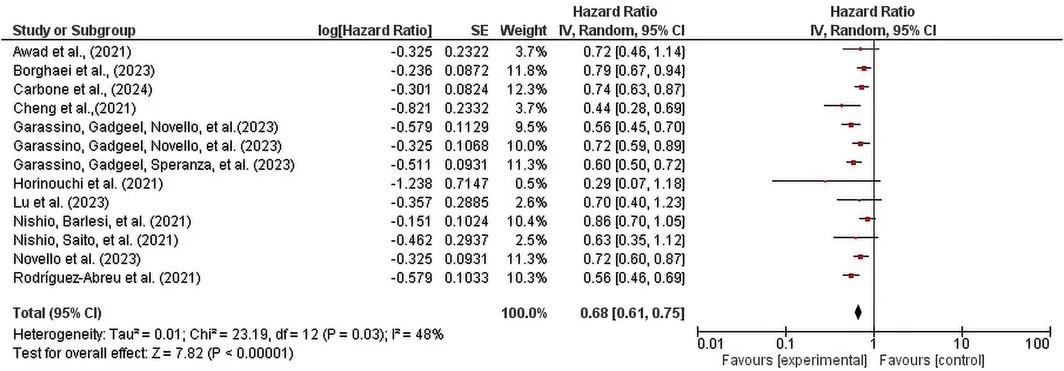
Forest plot relating ICI plus chemotherapy and chemotherapy for OS outcomes. The rows list incorporated studies/subgroups with reported log [HR], SE, study weight, and HR with 95% CIs. The *x* axis is logarithmic from 0.01 to 100; the vertical line at HR = 1 shows no difference between arms, and HR < 1 favors ICI. Pooled effect and heterogeneity statistics are also shown.

The forest plot shows the collective results of a meta‐analysis comparing ICI plus chemotherapy versus chemotherapy alone throughout 12 studies (Figure 9). Most individual studies revealed HRs < 1, representing a survival benefit that favors the combination therapy. This is further validated through the highly significant test for overall effect (*Z* = 7.82, *p* < 0.00001), favoring the conclusion that the addition of ICIs to chemotherapy offers higher clinical efficacy compared with chemotherapy alone. The overall pooled analysis showed a statistically significant reduction in the hazard of adverse outcomes with ICI plus chemotherapy (HR = 0.68 [95% CI: 0.75–0.61]). Although a few studies pointed out that CIs surpassed the threshold of no effect, the majority consistently supported the experimental arm. The heterogeneity among studies was moderate (*I* ^2^ = 48%) with some variability in study outcomes; however, it was satisfactory consistency overall.

###### 3.4.2.1.1 Subgroup Analyses for OS

**FIGURE 10:**
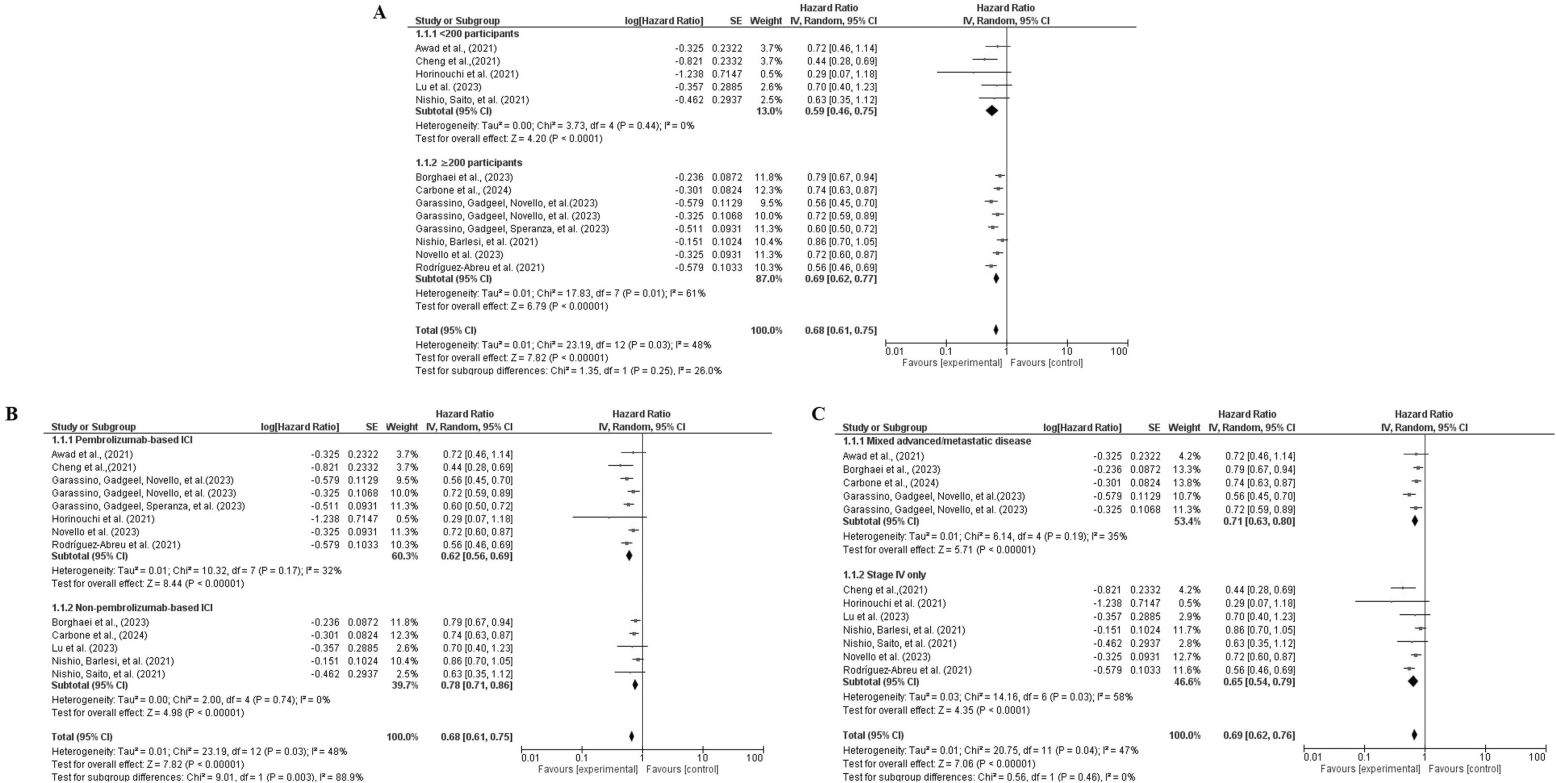
Forest plots for subgroup analysis of OS outcomes comparing ICI plus chemotherapy versus chemotherapy. HRs with 95% CIs (log [HR], SE, and study weight shown); *x*‐axis log scale 0.01–100 with 1 as the line of no effect. (A) By study size (≥ 200 vs. < 200), (B) by ICI agent (pembrolizumab‐based vs. nonpembrolizumab‐based), and (C) by disease stage (mixed advanced/metastatic NSCLC vs. Stage IV NSCLC).

The subgroup analyses measured the effect of combination therapy versus chemotherapy alone by study sample size, treatment type, and disease stage. Studies with < 200 participants showed a significant OS benefit for the combination (HR 0.59 [95% CI: 0.46–0.75]; *I* ^2^ = 0%, *p* = 0.44) showing consistent results throughout smaller RCTs. The studies with ≥ 200 participants also preferred the combination (HR 0.69 [95% CI: 0.62–0.77]) but displayed moderate heterogeneity (*I* ^2^ = 61%, *p* = 0.01) (Figure 10A). In treatment‐type subgroups, the pembrolizumab‐based ICI plus chemotherapy subgroup (eight studies) showed a significant survival advantage (HR 0.62 [95% CI: 0.56–0.69], *p* < 0.00001) with low‐to‐moderate heterogeneity (*I* ^2^ = 32%), whereas the nonpembrolizumab‐based subgroup (five studies) also showed a statistically significant benefit (HR 0.78 [95% CI: 0.71–0.86], *p* < 0.00001) with no heterogeneity (*I* ^2^ = 0%). The test for subgroup differences was statistically significant, representing greater benefit with pembrolizumab‐based combination therapy (*χ* ^2^ = 9.01, *p* = 0.003; *I* ^2^ = 88.9%) (Figure 10B). By disease stage, pooled analysis of five studies in mixed advanced/metastatic populations showed a significant OS improvement with the combination (HR 0.71 [95% CI: 0.63–0.80], *p* < 0.00001; *I* ^2^ = 35%), and seven Stage IV–only studies likewise supported the combination therapy (HR 0.65 [95% CI: 0.54–0.79], *p* < 0.0001; *I* ^2^ = 58%). The test for subgroup differences by stage was nonsignificant (*χ* ^2^ = 0.56, *p* = 0.46; *I* ^2^ = 0%), declaring advantage regardless of disease stage (Figure 10C).

###### 3.4.2.1.2 Publication Bias

**FIGURE 11:**
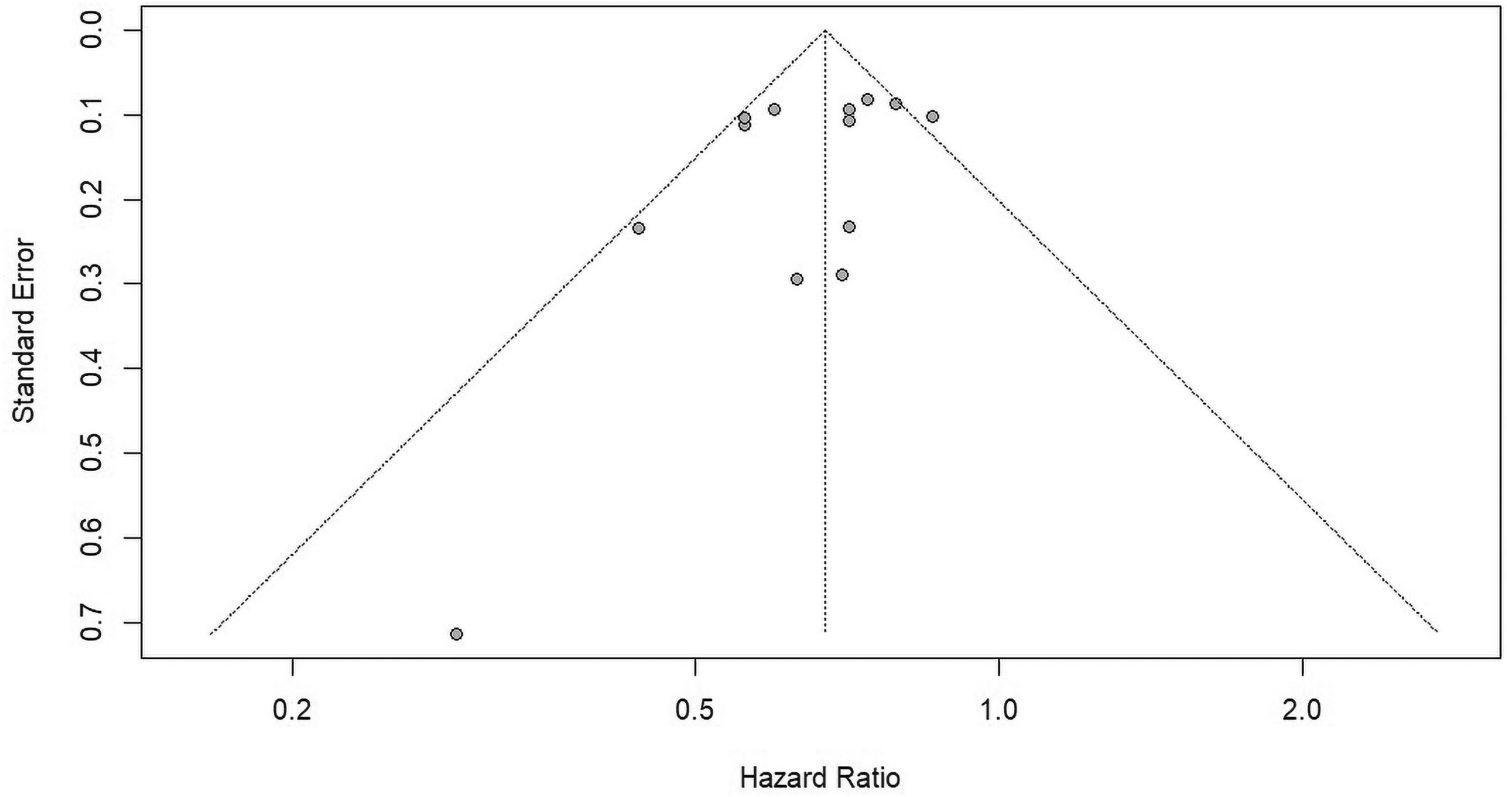
Funnel plot appraising publication bias analysis of OS comparing ICI plus chemotherapy versus chemotherapy, with HRs as the effect measure. Each dot signifies an individual study. The *x* axis shows HRs, and the *y* axis shows SE.

Visual assessment of the funnel plot showed a largely symmetrical distribution of studies around the pooled effect. Most studies collected near the top, representing acceptable accuracy (Figure 11). A small degree of asymmetry was noted, which was mainly driven by one study with a comparatively large SE. Egger's linear regression test shows statistically nonsignificant funnel plot asymmetry (*t* = −1.34, *p* = 0.2075), specifying no meaningful small‐study effects. Generally, there was no significant publication bias that could affect the meta‐analysis results. Therefore, the pooled estimates are likely reliable.

###### 3.4.2.1.3 Leave‐One‐Out Analysis

Leave‐one‐out sensitivity analysis was performed through using a random‐effects model (inverse variance method, DerSimonian‐Laird estimator for *τ* ^2^). It confirmed the strength of the pooled HR of 0.6731 (95% CI: 0.6105–0.7420, *p* < 0.0001, *τ* = 0.1153, *I* ^2^ = 47.6%) for ICI plus chemotherapy versus chemotherapy alone in NSCLC patients' OS (Supporting Information). Omitting individual studies produced HRs ranging from 0.6569 (95% CI: 0.5984–0.7210, [35]) to 0.6892 (95% CI: 0.6253–0.7597, [36]), with 95% CIs constantly < 1, that is, 0.5984–0.7597 and all significant (*p* < 0.0001). Heterogeneity remained moderate (*I* ^2^ = 36.6%–52.0%), with the lowest *τ* ^2^ (0.0088) upon excluding Nishio et al. [35] and the highest (0.0166) excluding Novello et al. [37]. No single study inexplicably influenced the estimate, showing stability crossways subsets.

##### 3.4.2.2 PFS Outcome

**FIGURE 12:**
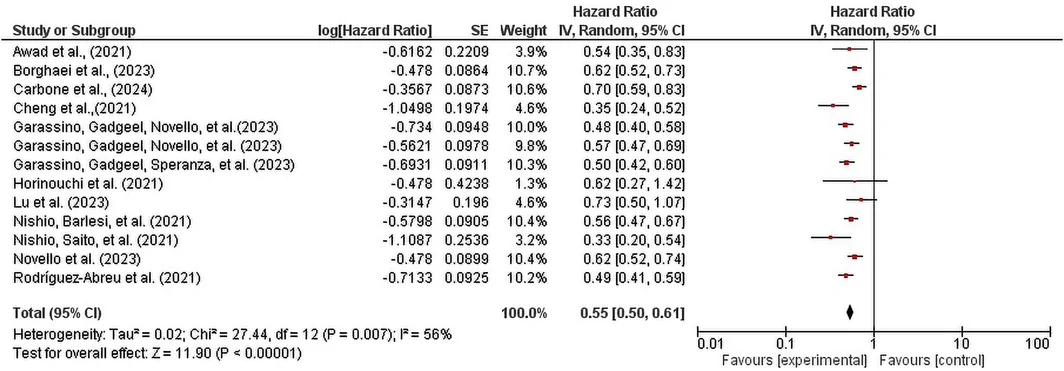
Forest plot comparing PFS outcomes between ICI plus chemotherapy and chemotherapy. The rows list included studies/subgroups with reported log [HR], SE, study weight, and HR with 95% CIs. The *x* axis is logarithmic from 0.01 to 100; the vertical line at HR = 1 indicates no difference between arms, and HR < 1 favors ICI. Pooled effect and heterogeneity statistics are also shown.

When looking at PFS outcomes in NSCLC patients, we basically noted it by comparing ICI together with chemotherapy versus chemotherapy alone. In the pooled analysis, we found that the combination therapy improved PFS outcomes significantly (HR = 0.55 [95% CI: 0.50–061], *p* < 0.00001), as it lowered the chance of disease progression or even death for patients. The heterogeneity in the studies seemed moderate (i.e., *I* ^2^ = 56%, *χ* ^2^ = 27.44, *p* = 0.007). These findings suggest that the adding of ICIs to chemotherapy gives a notable PFS benefit compared to chemotherapy alone (Figure 12).

###### 3.4.2.2.1 Subgroup Analysis for PFS

**FIGURE 13:**
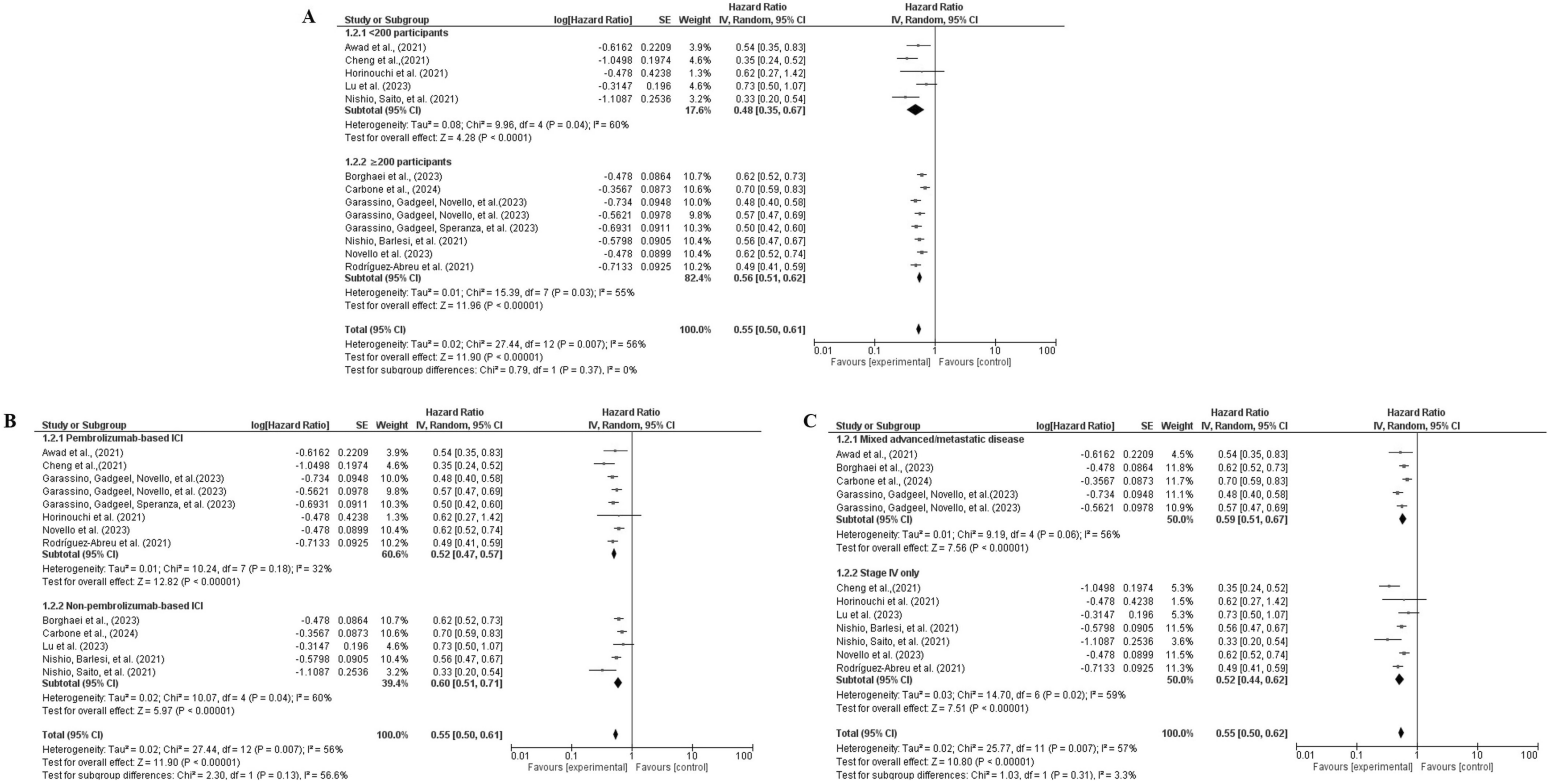
Forest plots subgroup analysis for PFS outcomes relating ICI plus chemotherapy versus chemotherapy. HRs with 95% CIs (log [HR], SE, and study weight shown); *x*‐axis log scale 0.01–100 with 1 as the line of no effect. (A) By study size (≥ 200 vs. < 200), (B) by ICI agent (pembrolizumab‐based vs. nonpembrolizumab‐based), and (C) by disease stage (mixed advanced/metastatic NSCLC vs. Stage IV NSCLC).

In a pooled subgroup analysis measuring PFS outcome, studies with < 200 participants, combination therapy significantly improved PFS outcome (HR 0.48 [95% CI: 0.35–0.67], *p* < 0.0001) with moderate heterogeneity (*I* ^2^ = 60%). This combination therapy also showed significant advantage (HR 0.56 [95% CI: 0.51–0.62], *p* < 0.00001) with moderate heterogeneity (*I* ^2^ = 55%) in studies with ≥ 200 participants. Hence, the overall pooled effect supported the experimental arm with no significant (*χ* ^2^ = 0.79, *p* = 0.37; *I* ^2^ = 0%) subgroup difference by population size (Figure 13A).

In the pembrolizumab‐based subgroup, the pooled HR was 0.52 (95% CI: 0.47–0.57) (*I* ^2^ = 32%), whereas the nonpembrolizumab‐based subgroup showed a pooled HR of 0.60 (95% CI: 0.51–0.71) (*I* ^2^ = 60%). This has turned out an overall pooled effect of HR 0.55 (95% CI: 0.50–0.61) with moderate heterogeneity (*I* ^2^ = 56%). However, no strong evidence of differing efficacy between pembrolizumab‐based and nonpembrolizumab‐based regimens was found owing to the test for subgroup differences was nonsignificant (*χ* ^2^ = 2.30, *p* = 0.13), representing (Figure 13B).

In disease stage–based subgroup analysis, combination therapy significantly enhanced PFS outcome in mixed advanced/metastatic populations (HR 0.59 [(95% CI: 0.51–0.67)], *p* < 0.00001; *I* ^2^ = 56%) and in Stage IV–only populations (HR 0.52 [95% CI: 0.44–0.62], *p* < 0.00001; *I* ^2^ = 59%). Largely, this paralleled to a 45% reduction in the risk of progression or death (HR 0.55 [95% CI: 0.50–0.62], *p* < 0.00001) with no significant subgroup difference by disease stage (*χ* ^2^ = 1.03, *p* = 0.31) (Figure 13C).

###### 3.4.2.2.2 Publication Bias

**FIGURE 14:**
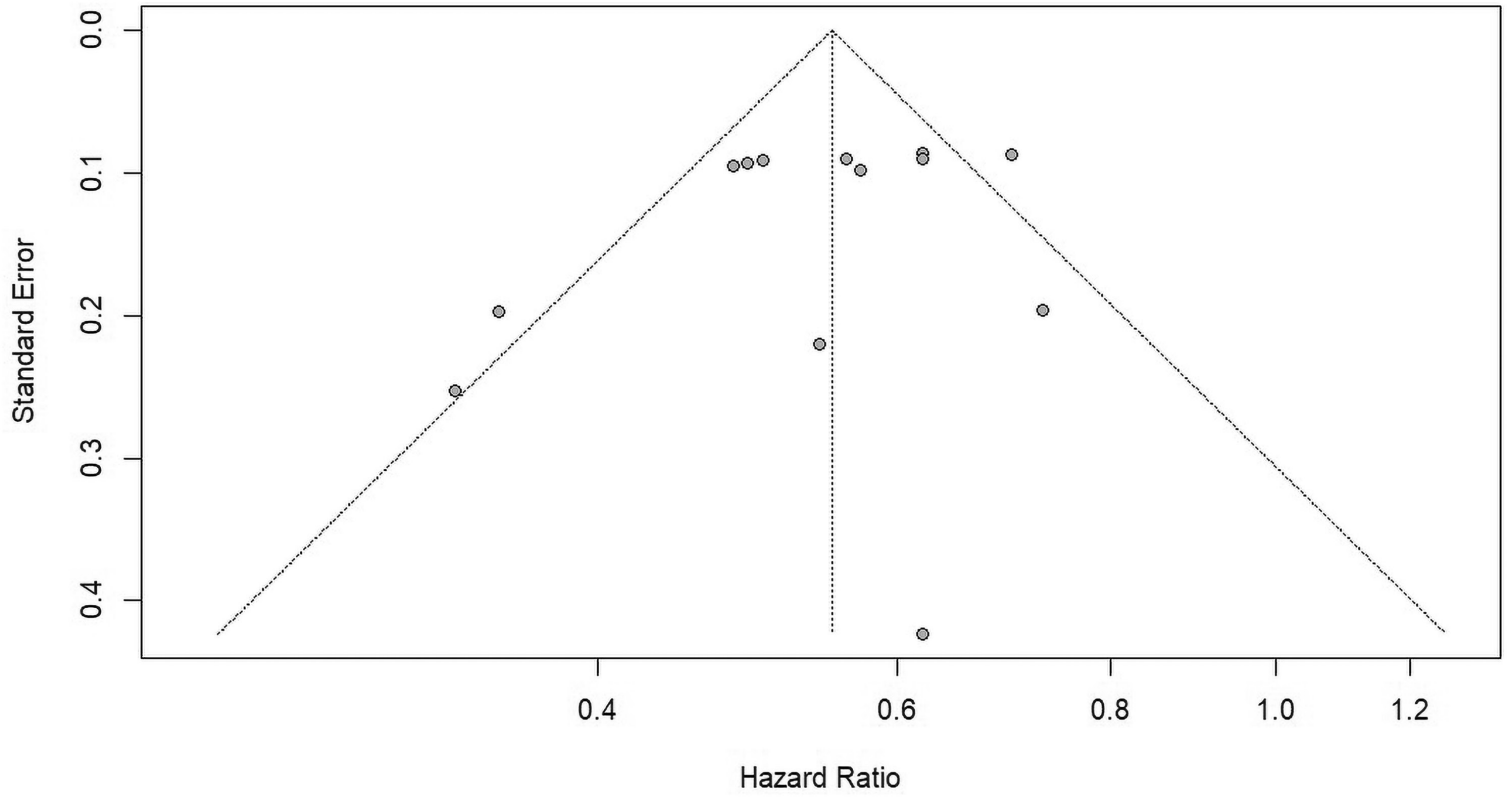
Funnel plot weighing publication bias analysis of PFS outcomes comparing ICI plus chemotherapy versus chemotherapy, with HRs as the effect measure. Each dot symbolizes a distinct study. The *x* axis shows HRs, and the *y* axis shows SE.

The funnel plot for the PFS outcome looked mostly symmetrical, with the studies scattered around the combined effect. Most studies fall within the 95% CIs, representing acceptable consistency (Figure 14). A slight asymmetry seemed visible, with a few studies scattered away from the central line. It might be reflecting moderate heterogeneity, some small study effects, and differences in the study setup and sample sizes rather than actual publication bias. Overall, the risk of publication bias was low.

###### 3.4.2.2.3 Leave‐One‐Out Analysis

For the leave‐one‐out sensitivity analysis, the overall pooled effect for PFS stayed stable, irrespective of which individual study got pulled out (Supporting Information). The HR stayed in the narrow window (0.5351–0.5617), and all corresponding CIs remained < 1. This implies that the beneficial effect of ICI plus chemotherapy was constantly preserved and was not driven by any single study. The analysis also suggests that no one trial had an unjustified impact on the pooled result. When each study was excluded in turn, the heterogeneity changed modestly (*I* ^2^ = 44.4%–59.9%, *τ* ^2^ = 0.1039–0.1414). However, the overall model showed *I* ^2^ of 56.3% and *τ* ^2^ of 0.1279. Although removing Carbone et al. [38] lowered heterogeneity, the direction and statistical significance of the pooled effect did not substantially change. Using an inverse‐variance approach with the DerSimonian‐Laird estimator, the outcomes stayed statistically significant. Thus, leave‐one‐out analysis strengthens the reliability by showing that the pooled PFS advantage stays robust even with the exclusion of any single study.

## 4 Discussion

This meta‐analysis shows that ICI‐based treatment improves both OS and PFS outcomes in advanced/metastatic NSCLC. The pooled estimates in this review were statistically significant for ICI monotherapy versus chemotherapy for both OS and PFS outcomes. However, the effect was stronger and more stable for ICI plus chemotherapy, especially for PFS outcome (pooled HR = 0.55 [95% CI: 0.50–0.61]), where the result stayed robust in subgroup, publication‐bias, and leave‐one‐out analyses. Our findings are parallel with large, RCT evidence, and recent syntheses showing resilient benefit from pembrolizumab‐based combination therapy. This is also in line with the study by Huang et al. [39], which found that pembrolizumab plus chemotherapy improved PFS (median 10.41 vs. 7.41 months, HR: 0.81, *p* = 0.02) and objective response rate (objective response rate [ORR]; RR: 1.74, 95% CI: 1.25–2.43) compared to pembrolizumab monotherapy in patients with PD‐L1 ≥ 50%. However, no statistically significant OS difference was observed in this subgroup (median 22.54 vs. 22.62 months, HR: 0.89, *p* = 0.24). In contrast, pembrolizumab plus chemotherapy provided a significant long‐term OS benefit specifically in patients with PD‐L1 1%–49% expression (median 20.88 vs. 13.60 months, HR: 0.77, *p* = 0.015). This suggests the combination is mainly advantageous for patients with PD‐L1 1%–49% expression, though it is go along with by increased treatment‐related adverse effects (trAEs) [39]. An important strength of this review is its separate analysis of ICI monotherapy from ICI combined with chemotherapy. In this way, it elucidates why the therapeutic effects fluctuate throughout ICI approaches. For instance, in the ICI monotherapy analysis, it conveyed benefits but presented greater heterogeneity and less steady PFS outcomes. On the other hand, the combination of pembrolizumab with chemotherapy showed a stronger advantage in PFS compared to chemotherapy alone crosswise RCTs. This pattern is biologically reasonable, as chemotherapy improves antitumor immune activity and provides earlier tumor cell reduction [40].

Regarding subgroup analysis, the findings helped elucidate some of the heterogeneity perceived through studies. Though pembrolizumab‐based combination therapy showed a statistically stronger pooled effect than nonpembrolizumab regimens, the subgroup variance for PFS was statistically nonsignificant in our analysis. This design is parallel with prior studies showing mixed subgroup signals, which likely reveal differences in histology, PD‐L1 status, line of therapy, stage distribution, and trial size rather than true loss of efficacy [39, 41, 42]. The heterogeneity in PFS outcome among NSCLC patients remains an important limitation as NSCLC is biologically heterogeneous and can affect therapeutic response [43, 44]. Moderate‐high *I* ^2^ values point out that the included RCTs were not seamlessly homogeneous, which is expected in NSCLC immunotherapy as RCTs vary in drug selection, chemotherapy strength, and disease stage [45]. Similar complications in therapeutic effects have been seen in other NSCLC immunotherapy meta‐analyses, especially when PD‐1/PD‐L1 monotherapy and chemo‐immunotherapy treatments were united within the same network [46, 47].

Beyond RCT‐level heterogeneity, biological resistance mechanisms also restrict the strength of ICI advantage. Although ICIs have brought some promising advances in the treatment of lung cancer, resistance remains the most significant challenge [48]. Therefore, all previous patients who showed initial signs of response to treatment experienced relapse, indicating that this lethal disease needs better biomarkers for predictive purposes along with novel therapeutic strategies [49]. The causes of resistance have been investigated to determine factors that may result in the failure of a particular therapy, such as alterations in the tumor's microenvironment and intrinsic characteristics of a tumor that might hinder immunologic perception [50]. Hence, research is currently being conducted to identify targets for immunotherapy other than the PD‐1/PD‐L1 axis [51]. So far, immunotherapy employed in small cell lung cancer (SCLC) has yet to gain momentum compared with NSCLC [52]. Although ICIs have shown some promise in SCLC, their effect on OS when combined with chemotherapy in advanced‐stage is less dramatic than that observed in NSCLC cases [53]. Hence, it highlights the necessity for future studies aimed at the optimization of immunotherapy for SCLC and the identification of the biological differences between SCLC and NSCLC [54]. In clinical practice, it have opened new avenues for the treatment of advanced NSCLC with better survival outcomes and have given patients an option outside conventional chemotherapy [55]. Together, these descriptions underscore the strength of the evidence and set the stage for sensitivity analyses.

The leave‐one‐out effects reinforce the standing of the review because the pooled effect remained stable after removing each study one by one. It is clear that no single RCT, even with large population size, was influencing the conclusion. Hence, this type of robustness is consistent with potentially good quality meta‐analyses in oncology, where stable pooled estimates are inferred as resilient signals for clinical use [56]. Overall, the discussion can be concluded that the data supports ICI plus chemotherapy as the most reliable and consistent first‐line approach for advanced/metastatic NSCLC when compared with chemotherapy alone, with a stable PFS benefit and significant OS improvement. At the same time, the changeability seen in ICI monotherapy studies and in subgroup analyses proves that not all ICI approaches perform equally through all populations and that is why disease stage, therapeutic regimen, and RCT design remain important when construing the literature [57].

## 5 Limitations

The main limitations of this meta‐analysis are the moderate to high heterogeneity through many trials, especially for PFS outcome, the inconstant risk of bias among the included studies, and the clinical diversity in immunotherapeutic agents, chemotherapy strengths, disease stage, and so on. In addition, this review depends on a comparatively small number of studies for some subgroup analyses, and hence, it restricts the accuracy of those estimates and makes publication‐bias assessment less consistent. The study also used study‐level combined data instead of individual patient data, which limits correction for important predictive factors such as PD‐L1 status, histology, and erstwhile treatment differences.

Furthermore, this review study did not evaluate molecular profiling outside PD‐L1 expression. This limitation has occurred because all studies with molecular biomarkers in the same meta‐analysis would entail a diverse design, which was outside the scope and objectives of this study. Additionally, the heterogeneous reporting throughout the included studies and inadequate toxicity‐related data in some studies restricted the systematic appraisal of therapeutic‐based adverse events. This limitation has also constrained the potential to compare immunotherapy and chemotherapy safety profiles in a quantitative way. Finally, this limitation will restrict conclusions apropos the risk–benefit ratio in specific clinical subgroups.

## 6 Conclusion

This meta‐analysis shows that ICI interventions (alone or in combination) provide strong advantages, in terms of OS and PFS outcomes, over chemotherapy alone for adults with advanced/metastatic NSCLC. Both types of interventions showed advantages throughout wide‐ranging study populations, with stronger effects in smaller RCTs and pembrolizumab‐based treatments. Though results held strongly across larger studies, different disease stages, and various ICI types, high heterogeneity in some analyses highlights variability in the study designs and patient characteristics. Additionally, sensitivity analyses confirmed the constancy of findings, with minimal publication bias. Risk of bias was raised in the overall analysis due to concerns in randomization, intervention deviations, and outcome handling. Hence, it justifies cautious interpretation and explanation while emphasizing ICI interventions as the preferable first‐line choice in this setting.

## Author Contributions

Yingjie Jiao and Yan Zhao confirm the responsibility for all aspects of this meta‐analysis review article, including study conception and design, data collection, analysis, and manuscript preparation. Both the authors reviewed the whole article and approved the final version of the manuscript.

## Funding

The authors have nothing to report.

## Conflicts of Interest

The authors declare no conflicts of interest.

## Supporting information


**Data S1:** Supporting Information.

## Data Availability

The datasets supporting the conclusions of this study are available on reasonable request to the corresponding author.
