# Supplementary material for: Advancements in Immunotherapy for Lung Cancer: A Meta‐Analysis of Clinical Trial Outcomes
Source: Clin Respir J. 2026 Sep 9;20(9):e70223. doi: 10.1111/crj.70223 (PMC13557920; doi:10.1111/crj.70223)
Supplement: Supplementary file 1 [file CRJ-20-e70223-s001.docx]

**Supplementary Data**

**QUERIES**

**PubMed:**(("Nivolumab"[Mesh] OR "Atezolizumab"[Mesh] OR "Pembrolizumab"[Mesh] OR nivolumab[tiab] OR atezolizumab[tiab] OR pembrolizumab[tiab])) AND ( ("Carcinoma, Non-Small-Cell Lung"[Mesh] OR "non small cell lung cancer"[tiab] OR NSCLC[tiab])) AND (("Survival"[Mesh] OR "overall survival"[tiab] OR "progression-free survival"[tiab] OR PFS[tiab])) AND (("Antineoplastic Combined Chemotherapy Protocols"[Mesh] OR chemotherapy[tiab] OR "chemotherapeutic agents"[tiab] OR "chemotherapy regimen"[tiab]))

**SCOPUS:** TITLE-ABS-KEY((nivolumab OR atezolizumab OR pembrolizumab) AND ("non small cell lung cancer" OR NSCLC) AND ("overall survival" OR "progression free survival" OR PFS) AND (chemotherapy OR "chemotherapeutic agents" OR "chemotherapy regimen"))

**WOS**: TS= ((nivolumab OR atezolizumab OR pembrolizumab) AND ("non small cell lung cancer" OR NSCLC) AND ("overall survival" OR "progression free survival" OR PFS) AND (chemotherapy OR "chemotherapeutic agents" OR "chemotherapy regimen"))

**PRISMA**

A) **IDENTIFICATION**

**Total Records = 7849**

- PubMed = 1120
- SCOPUS = 4312
- WOS =2417

**Duplicates** = 2571+36

**Total Records without duplicates** = 5242

**Records (2021-24)** = 2780

**Records (English)** = 2758

**B) TITLE AND ABSTRACT SCREENING**

**Records eligible for title and abstract screening** = 2758

**Records excluded after title and abstract screening** = 2736

**Records included after title and abstract screening** = 22

**Records eligible for full text screening = 22**

**C) FULL TEXT SCREENING**

**Records excluded after full text screening = 1**

- Wrong study design = 1 (Fan et al., 2023)

**Records included after full text screening = 21** (Awad et al., 2021), (Baramidze et al., 2024), (Borghaei et al., 2023), (Carbone et al., 2024), (Cheng et al., 2021), (de Castro Jr et al., 2023), (Gadgeel et al., 2022), (Garassino, Gadgeel, Novello, et al., 2023), (Garassino, Gadgeel, Speranza, et al., 2023), (Horinouchi et al., 2021), (Jassem et al., 2021), (Lu et al., 2023), (Nishio, Barlesi, et al., 2021), (Nishio, Saito, et al., 2021), (Novello et al., 2023), (Reck et al., 2024), (Reck et al., 2021), (Rodríguez-Abreu et al., 2021), (Satouchi et al., 2021), (Sun et al., 2023), (Wu et al., 2021)

**Records excluded due to insufficient statistical data = 4** (Baramidze et al., 2024), (Gadgeel et al., 2022), (Reck et al., 2024), (Sun et al., 2023)

**Records eligible for meta - analysis = 17**

**References**

Awad, M. M., Gadgeel, S. M., Borghaei, H., Patnaik, A., Yang, J. C.-H., Powell, S. F., Gentzler, R. D., Martins, R. G., Stevenson, J. P., & Altan, M. (2021). Long-term overall survival from KEYNOTE-021 cohort G: pemetrexed and carboplatin with or without pembrolizumab as first-line therapy for advanced nonsquamous NSCLC. *Journal of thoracic oncology*, *16*(1), 162-168.

Baramidze, A., Makharadze, T., Gogishvili, M., Melkadze, T., Giorgadze, D., Penkov, K., Laktionov, K., Nemsadze, G., Nechaeva, M., & Rozhkova, I. (2024). Cemiplimab plus chemotherapy versus chemotherapy alone in non-small cell lung cancer with PD-L1≥ 1%: A subgroup analysis from the EMPOWER-Lung 3 part 2 trial. *Lung Cancer*, *193*, 107821.

Borghaei, H., O’Byrne, K., Paz-Ares, L., Ciuleanu, T.-E., Yu, X., Pluzanski, A., Nagrial, A., Havel, L., Kowalyszyn, R., & Valette, C. (2023). Nivolumab plus chemotherapy in first-line metastatic non-small-cell lung cancer: results of the phase III CheckMate 227 Part 2 trial. *ESMO open*, *8*(6), 102065.

Carbone, D. P., Ciuleanu, T.-E., Schenker, M., Cobo, M., Bordenave, S., Juan-Vidal, O., Menezes, J., Reinmuth, N., Richardet, E., & Cheng, Y. (2024). Four-year clinical update and treatment switching-adjusted outcomes with first-line nivolumab plus ipilimumab with chemotherapy for metastatic non-small cell lung cancer in the CheckMate 9LA randomized trial. *Journal for Immunotherapy of Cancer*, *12*(2), e008189.

Cheng, Y., Zhang, L., Hu, J., Wang, D., Hu, C., Zhou, J., Wu, L., Cao, L., Liu, J., & Zhang, H. (2021). Pembrolizumab plus chemotherapy for Chinese patients with metastatic squamous NSCLC in KEYNOTE-407. *JTO clinical and research reports*, *2*(10), 100225.

de Castro Jr, G., Kudaba, I., Wu, Y.-L., Lopes, G., Kowalski, D. M., Turna, H. Z., Caglevic, C., Zhang, L., Karaszewska, B., & Laktionov, K. K. (2023). Five-year outcomes with pembrolizumab versus chemotherapy as first-line therapy in patients with non–small-cell lung cancer and programmed death ligand-1 tumor proportion score≥ 1% in the KEYNOTE-042 study. *Journal of clinical oncology*, *41*(11), 1986-1991.

Fan, Y., Liu, Y., Wang, L., Cai, Y., Cao, W., Sun, W., Zou, X., Li, B., Zhang, Z., & Cai, S. (2023). bITH, a blood-based metric of intratumor heterogeneity, is associated with clinical response to immune checkpoint blockade in non-small cell lung cancer. *EBioMedicine*, *91*.

Gadgeel, S., Gray, J. E., Rizzo, M. T., Peterson, P., Kim, J. S., & Rodríguez-Abreu, D. (2022). Pemetrexed and platinum plus pembrolizumab in patients with metastatic nonsquamous NSCLC by tumor burden at baseline: a post hoc efficacy analysis of KEYNOTE-189. *JTO clinical and research reports*, *3*(11), 100389.

Garassino, M. C., Gadgeel, S., Novello, S., Halmos, B., Felip, E., Speranza, G., Hui, R., Garon, E. B., Horinouchi, H., & Sugawara, S. (2023). Associations of tissue tumor mutational burden and mutational status with clinical outcomes with pembrolizumab plus chemotherapy versus chemotherapy for metastatic NSCLC. *JTO clinical and research reports*, *4*(1), 100431.

Garassino, M. C., Gadgeel, S., Speranza, G., Felip, E., Esteban, E., Dómine, M., Hochmair, M. J., Powell, S. F., Bischoff, H. G., & Peled, N. (2023). Pembrolizumab plus pemetrexed and platinum in nonsquamous non–small-cell lung cancer: 5-year outcomes from the phase 3 KEYNOTE-189 study. *Journal of clinical oncology*, *41*(11), 1992-1998.

Horinouchi, H., Nogami, N., Saka, H., Nishio, M., Tokito, T., Takahashi, T., Kasahara, K., Hattori, Y., Ichihara, E., & Adachi, N. (2021). Pembrolizumab plus pemetrexed‐platinum for metastatic nonsquamous non–small‐cell lung cancer: KEYNOTE‐189 Japan Study. *Cancer science*, *112*(8), 3255-3265.

Jassem, J., de Marinis, F., Giaccone, G., Vergnenegre, A., Barrios, C. H., Morise, M., Felip, E., Oprean, C., Kim, Y.-C., & Andric, Z. (2021). Updated overall survival analysis from IMpower110: atezolizumab versus platinum-based chemotherapy in treatment-naive programmed death-ligand 1–selected NSCLC. *Journal of thoracic oncology*, *16*(11), 1872-1882.

Lu, S., Fang, J., Wang, Z., Fan, Y., Liu, Y., He, J., Zhou, J., Hu, J., Xia, J., & Liu, W. (2023). Results from the IMpower132 China cohort: Atezolizumab plus platinum‐based chemotherapy in advanced non‐small cell lung cancer. *Cancer Medicine*, *12*(3), 2666-2676.

Nishio, M., Barlesi, F., West, H., Ball, S., Bordoni, R., Cobo, M., Longeras, P. D., Goldschmidt Jr, J., Novello, S., & Orlandi, F. (2021). Atezolizumab plus chemotherapy for first-line treatment of nonsquamous NSCLC: results from the randomized phase 3 IMpower132 trial. *Journal of thoracic oncology*, *16*(4), 653-664.

Nishio, M., Saito, H., Goto, K., Watanabe, S., Sueoka‐Aragane, N., Okuma, Y., Kasahara, K., Chikamori, K., Nakagawa, Y., & Kawakami, T. (2021). IMpower132: Atezolizumab plus platinum‐based chemotherapy vs chemotherapy for advanced NSCLC in Japanese patients. *Cancer science*, *112*(4), 1534-1544.

Novello, S., Kowalski, D. M., Luft, A., Gümüş, M., Vicente, D., Mazières, J., Rodríguez-Cid, J., Tafreshi, A., Cheng, Y., & Lee, K. H. (2023). Pembrolizumab plus chemotherapy in squamous non–small-cell lung cancer: 5-year update of the phase III KEYNOTE-407 study. *Journal of clinical oncology*, *41*(11), 1999-2006.

Reck, M., Barlesi, F., Yang, J. C.-H., Westeel, V., Felip, E., Özgüroğlu, M., Dols, M. C., Sullivan, R., Kowalski, D. M., & Andric, Z. (2024). Avelumab versus platinum-based doublet chemotherapy as first-line treatment for patients with high-expression programmed death-ligand 1–positive metastatic NSCLC: primary analysis from the phase 3 JAVELIN lung 100 trial. *Journal of thoracic oncology*, *19*(2), 297-313.

Reck, M., Rodríguez-Abreu, D., Robinson, A. G., Hui, R., Csőszi, T., Fülöp, A., Gottfried, M., Peled, N., Tafreshi, A., & Cuffe, S. (2021). Five-year outcomes with pembrolizumab versus chemotherapy for metastatic non–small-cell lung cancer with PD-L1 tumor proportion score≥ 50%. *Journal of clinical oncology*, *39*(21), 2339-2349.

Rodríguez-Abreu, D., Powell, S., Hochmair, M., Gadgeel, S., Esteban, E., Felip, E., Speranza, G., De Angelis, F., Dómine, M., & Cheng, S. (2021). Pemetrexed plus platinum with or without pembrolizumab in patients with previously untreated metastatic nonsquamous NSCLC: protocol-specified final analysis from KEYNOTE-189. *Annals of Oncology*, *32*(7), 881-895.

Satouchi, M., Nosaki, K., Takahashi, T., Nakagawa, K., Aoe, K., Kurata, T., Sekine, A., Horiike, A., Fukuhara, T., & Sugawara, S. (2021). First‐line pembrolizumab vs chemotherapy in metastatic non‐small‐cell lung cancer: KEYNOTE‐024 Japan subset. *Cancer science*, *112*(12), 5000-5010.

Sun, D., Liu, J., Zhou, H., Shi, M., Sun, J., Zhao, S., Chen, G., Zhang, Y., Zhou, T., & Ma, Y. (2023). Classification of tumor immune microenvironment according to programmed death-ligand 1 expression and immune infiltration predicts response to immunotherapy plus chemotherapy in advanced patients with NSCLC. *Journal of thoracic oncology*, *18*(7), 869-881.

Wu, Y.-L., Zhang, L., Fan, Y., Zhou, J., Zhou, Q., Li, W., Hu, C., Chen, G., Zhang, X., & Zhou, C. (2021). Randomized clinical trial of pembrolizumab vs chemotherapy for previously untreated Chinese patients with PD-L1-positive locally advanced or metastatic non-small-cell lung cancer: KEYNOTE-042 China Study. In.

**LEAVE ONE OUT**

(ICI vs. Chemotherapy, OS outcome)

summary(loo)

Leave-one-out meta-analysis

HR	95%-CI p-value

Omitting de Castro Jr et al. (2023) 0.6905 [0.5548; 0.8593]	0.0009

Omitting Jassem et al. (2021)	0.6913 [0.5759; 0.8299] < 0.0001

Omitting Reck et al. (2021)	0.7683 [0.6672; 0.8847]	0.0003

Omitting Satouchi et al. (2021)	0.7524 [0.6638; 0.8529] < 0.0001

Omitting Wu et al. (2021)	0.7418 [0.6246; 0.8809]	0.0007

Random effects model	0.7335 [0.6354; 0.8466] < 0.0001

tau^2	tau	I^2

Omitting de Castro Jr et al. (2023) 0.0235 0.1532 50.5%

Omitting Jassem et al. (2021)	0.0152 0.1233 46.3%

Omitting Reck et al. (2021)	0.0067 0.0818 31.9%

Omitting Satouchi et al. (2021)	0.0057 0.0756 34.5%

Omitting Wu et al. (2021)	0.0145 0.1204 51.8%

Random effects model	0.0107 0.1034 43.1%

Details of meta-analysis methods:

- Inverse variance method

- DerSimonian-Laird estimator for tau^2

- Calculation of I^2 based on Q

**LEAVE ONE OUT**

(ICI vs. Chemotherapy, PFS outcome)

>	summary(loo)

Leave-one-out meta-analysis

HR	95%-CI p-value

Omitting de Castro Jr et al. (2023) 0.6043 [0.4091; 0.8928] 0.0114

Omitting Jassem et al. (2021)	0.6536 [0.4088; 1.0449] 0.0756

Omitting Reck et al. (2021)	0.7722 [0.5576; 1.0694] 0.1197

Omitting Satouchi et al. (2021)	0.7853 [0.5750; 1.0725] 0.1286

Omitting Wu et al. (2021)	0.6109 [0.4029; 0.9263] 0.0203

Random effects model	0.6874 [0.4913; 0.9618] 0.0287

tau^2	tau	I^2

Omitting de Castro Jr et al. (2023) 0.1232 0.3510 86.0%

Omitting Jassem et al. (2021)	0.1918 0.4379 92.0%

Omitting Reck et al. (2021)	0.0831 0.2883 87.1%

Omitting Satouchi et al. (2021)	0.0894 0.2990 90.2%

Omitting Wu et al. (2021)	0.1486 0.3854 92.6%

Random effects model	0.1192 0.3453 90.5%

Details of meta-analysis methods:

- Inverse variance method

- DerSimonian-Laird estimator for tau^2

- Calculation of I^2 based on Q

**LEAVE ONE OUT**

(ICI plus Chemotherapy vs. Chemotherapy, OS outcome)

> summary(loo)

Leave-one-out meta-analysis

HR

Omitting Awad et al. (2021)	0.6708

Omitting Borghaei et al. (2023)	0.6590

Omitting Carbone et al. (2024)	0.6629

Omitting Cheng et al. (2021)	0.6848

Omitting Garassino, Gadgeel, Novello et al. (2023a) 0.6873

Omitting Garassino, Gadgeel, Novello et al. (2023b) 0.6675

Omitting Garassino, Gadgeel, Speranza et al. (2023) 0.6828

Omitting Horinouchi et al. (2021)	0.6760

Omitting Lu et al. (2023)	0.6716

Omitting Nishio, Barlesi et al. (2021)	0.6569

Omitting Nishio, Saito et al. (2021)	0.6735

Omitting Novello et al. (2023)	0.6668

Omitting Rodriguez-Abreu et al. (2021)	0.6892

Random effects model	0.6731

95%-CI

Omitting Awad et al. (2021)	[0.6054; 0.7432]

Omitting Borghaei et al. (2023)	[0.5948; 0.7302]

Omitting Carbone et al. (2024)	[0.5942; 0.7395]

Omitting Cheng et al. (2021)	[0.6240; 0.7517]

Omitting Garassino, Gadgeel, Novello et al. (2023a) [0.6228; 0.7586]

Omitting Garassino, Gadgeel, Novello et al. (2023b) [0.5988; 0.7440]

Omitting Garassino, Gadgeel, Speranza et al. (2023) [0.6152; 0.7578]

Omitting Horinouchi et al. (2021)	[0.6136; 0.7447]

Omitting Lu et al. (2023)	[0.6066; 0.7435]

Omitting Nishio, Barlesi et al. (2021)	[0.5984; 0.7210]

Omitting Nishio, Saito et al. (2021)	[0.6085; 0.7455]

Omitting Novello et al. (2023)	[0.5973; 0.7443]

Omitting Rodriguez-Abreu et al. (2021)	[0.6253; 0.7597]

Random effects model	[0.6105; 0.7420]

p-value tau^2

Omitting Awad et al. (2021)	< 0.0001 0.0148

Omitting Borghaei et al. (2023)	< 0.0001 0.0126

Omitting Carbone et al. (2024)	< 0.0001 0.0159

Omitting Cheng et al. (2021)	< 0.0001 0.0103

Omitting Garassino, Gadgeel, Novello et al. (2023a) < 0.0001 0.0114

Omitting Garassino, Gadgeel, Novello et al. (2023b) < 0.0001 0.0161

Omitting Garassino, Gadgeel, Speranza et al. (2023) < 0.0001 0.0136

Omitting Horinouchi et al. (2021)	< 0.0001 0.0128

Omitting Lu et al. (2023)	< 0.0001 0.0147

Omitting Nishio, Barlesi et al. (2021)	< 0.0001 0.0088

Omitting Nishio, Saito et al. (2021)	< 0.0001 0.0146

Omitting Novello et al. (2023)	< 0.0001 0.0166

Omitting Rodriguez-Abreu et al. (2021)	< 0.0001 0.0106

Random effects model	< 0.0001 0.0133

tau	I^2

Omitting Awad et al. (2021)	0.1218 51.9%

Omitting Borghaei et al. (2023)	0.1124 44.4%

Omitting Carbone et al. (2024)	0.1261 49.6%

Omitting Cheng et al. (2021)	0.1016 42.9%

Omitting Garassino, Gadgeel, Novello et al. (2023a) 0.1068 43.4%

Omitting Garassino, Gadgeel, Novello et al. (2023b) 0.1269 51.7%

Omitting Garassino, Gadgeel, Speranza et al. (2023) 0.1166 46.7%

Omitting Horinouchi et al. (2021)	0.1130 48.8%

Omitting Lu et al. (2023)	0.1214 52.0%

Omitting Nishio, Barlesi et al. (2021)	0.0936 36.6%

Omitting Nishio, Saito et al. (2021)	0.1210 51.8%

Omitting Novello et al. (2023)	0.1288 51.6%

Omitting Rodriguez-Abreu et al. (2021)	0.1031 41.3%

Random effects model	0.1153 47.6%

Details of meta-analysis methods:

- Inverse variance method

- DerSimonian-Laird estimator for tau^2

- Calculation of I^2 based on Q

**LEAVE ONE OUT**

(ICI plus Chemotherapy vs. Chemotherapy, PFS outcome)

> summary(loo)

Leave-one-out meta-analysis

HR

Omitting Awad et al. (2021)	0.5487

Omitting Borghaei et al. (2023)	0.5404

Omitting Carbone et al. (2024)	0.5351

Omitting Cheng et al. (2021)	0.5617

Omitting Garassino, Gadgeel, Novello et al. (2023a) 0.5572

Omitting Garassino, Gadgeel, Novello et al. (2023b) 0.5455

Omitting Garassino, Gadgeel, Speranza et al. (2023) 0.5542

Omitting Horinouchi et al. (2021)	0.5476

Omitting Lu et al. (2023)	0.5416

Omitting Nishio, Barlesi et al. (2021)	0.5463

Omitting Nishio, Saito et al. (2021)	0.5586

Omitting Novello et al. (2023)	0.5406

Omitting Rodriguez-Abreu et al. (2021)	0.5557

Random effects model	0.5489

95%-CI

Omitting Awad et al. (2021)	[0.4948; 0.6086]

Omitting Borghaei et al. (2023)	[0.4851; 0.6019]

Omitting Carbone et al. (2024)	[0.4873; 0.5875]

Omitting Cheng et al. (2021)	[0.5124; 0.6158]

Omitting Garassino, Gadgeel, Novello et al. (2023a) [0.5021; 0.6183]

Omitting Garassino, Gadgeel, Novello et al. (2023b) [0.4887; 0.6088]

Omitting Garassino, Gadgeel, Speranza et al. (2023) [0.4978; 0.6169]

Omitting Horinouchi et al. (2021)	[0.4947; 0.6061]

Omitting Lu et al. (2023)	[0.4899; 0.5987]

Omitting Nishio, Barlesi et al. (2021)	[0.4890; 0.6104]

Omitting Nishio, Saito et al. (2021)	[0.5085; 0.6137]

Omitting Novello et al. (2023)	[0.4854; 0.6021]

Omitting Rodriguez-Abreu et al. (2021)	[0.4999; 0.6176]

Random effects model	[0.4972; 0.6059]

p-value tau^2

Omitting Awad et al. (2021)	< 0.0001 0.0178

Omitting Borghaei et al. (2023)	< 0.0001 0.0182

Omitting Carbone et al. (2024)	< 0.0001 0.0108

Omitting Cheng et al. (2021)	< 0.0001 0.0117

Omitting Garassino, Gadgeel, Novello et al. (2023a) < 0.0001 0.0163

Omitting Garassino, Gadgeel, Novello et al. (2023b) < 0.0001 0.0196

Omitting Garassino, Gadgeel, Speranza et al. (2023) < 0.0001 0.0179

Omitting Horinouchi et al. (2021)	< 0.0001 0.0175

Omitting Lu et al. (2023)	< 0.0001 0.0159

Omitting Nishio, Barlesi et al. (2021)	< 0.0001 0.0200

Omitting Nishio, Saito et al. (2021)	< 0.0001 0.0130

Omitting Novello et al. (2023)	< 0.0001 0.0182

Omitting Rodriguez-Abreu et al. (2021)	< 0.0001 0.0172

Random effects model	< 0.0001 0.0164

tau	I^2

Omitting Awad et al. (2021)	0.1335 59.9%

Omitting Borghaei et al. (2023)	0.1349 57.4%

Omitting Carbone et al. (2024)	0.1039 44.4%

Omitting Cheng et al. (2021)	0.1081 49.3%

Omitting Garassino, Gadgeel, Novello et al. (2023a) 0.1278 55.3%

Omitting Garassino, Gadgeel, Novello et al. (2023b) 0.1398 59.9%

Omitting Garassino, Gadgeel, Speranza et al. (2023) 0.1339 57.3%

Omitting Horinouchi et al. (2021)	0.1322 59.9%

Omitting Lu et al. (2023)	0.1261 57.0%

Omitting Nishio, Barlesi et al. (2021)	0.1414 59.9%

Omitting Nishio, Saito et al. (2021)	0.1141 52.3%

Omitting Novello et al. (2023)	0.1348 57.6%

Omitting Rodriguez-Abreu et al. (2021)	0.1310 56.4%

Random effects model	0.1279 56.3%

Details of meta-analysis methods:

- Inverse variance method

- DerSimonian-Laird estimator for tau^2

- Calculation of I^2 based on Q
